# Glucotypes reveal new patterns of glucose dysregulation

**DOI:** 10.1371/journal.pbio.2005143

**Published:** 2018-07-24

**Authors:** Heather Hall, Dalia Perelman, Alessandra Breschi, Patricia Limcaoco, Ryan Kellogg, Tracey McLaughlin, Michael Snyder

**Affiliations:** 1 Stanford University, Stem Cell Biology and Regenerative Medicine, Stanford, California, United States of America; 2 Stanford University, Department of Genetics, Stanford, California, United States of America; 3 Stanford University, Department of Medicine, Division of Endocrinology, Stanford, California, United States of America; Duke University, United States of America

## Abstract

Diabetes is an increasing problem worldwide; almost 30 million people, nearly 10% of the population, in the United States are diagnosed with diabetes. Another 84 million are prediabetic, and without intervention, up to 70% of these individuals may progress to type 2 diabetes. Current methods for quantifying blood glucose dysregulation in diabetes and prediabetes are limited by reliance on single-time-point measurements or on average measures of overall glycemia and neglect glucose dynamics. We have used continuous glucose monitoring (CGM) to evaluate the frequency with which individuals demonstrate elevations in postprandial glucose, the types of patterns, and how patterns vary between individuals given an identical nutrient challenge. Measurement of insulin resistance and secretion highlights the fact that the physiology underlying dysglycemia is highly variable between individuals. We developed an analytical framework that can group individuals according to specific patterns of glycemic responses called “glucotypes” that reveal heterogeneity, or subphenotypes, within traditional diagnostic categories of glucose regulation. Importantly, we found that even individuals considered normoglycemic by standard measures exhibit high glucose variability using CGM, with glucose levels reaching prediabetic and diabetic ranges 15% and 2% of the time, respectively. We thus show that glucose dysregulation, as characterized by CGM, is more prevalent and heterogeneous than previously thought and can affect individuals considered normoglycemic by standard measures, and specific patterns of glycemic responses reflect variable underlying physiology. The interindividual variability in glycemic responses to standardized meals also highlights the personal nature of glucose regulation. Through extensive phenotyping, we developed a model for identifying potential mechanisms of personal glucose dysregulation and built a webtool for visualizing a user-uploaded CGM profile and classifying individualized glucose patterns into glucotypes.

## Author summary

One in 10 individuals is affected by diabetes, a condition involving abnormal regulation of blood glucose. Currently, diabetes is assessed using single-time or average measurements of blood glucose, without consideration for how blood glucose fluctuates over time. We used continuous glucose monitoring (CGM) technology to evaluate how blood glucose fluctuates in individuals over time. We found that many individuals considered nondiabetic by standard measures, in fact, experienced frequent elevations in blood glucose levels into the impaired glucose-tolerant or diabetic range. We developed a model for determining the “glucotype” of an individual, a more comprehensive measure of the pattern of glucose excursions than the standard laboratory tests in current use. We also built a web tool for interactively visualizing an individual’s glucose profile and performing glucotype assessment. With greater adoption of CGM technology, glucotype assessment may become an important tool in early identification of those at risk for type 2 diabetes and/or cardiovascular disease.

## Introduction

Type 2 diabetes is one of the most significant health problems worldwide, affecting 30.2 million adults in the US [1] and 422 million worldwide [2], with global costs in excess of $825 billion [3]. In the US alone, there are 84 million individuals with prediabetes, which converts to type 2 diabetes with an annual rate of approximately 10%. Type 2 diabetes is genetically heterogeneous. Increasing data suggest that it is also physiologically heterogeneous [4]. Blood glucose levels are regulated by coordinated physiological responses of multiple organs to ensure adequate energy delivery to cells throughout the body. Glucose disposal into cells is, in part, mediated by insulin, and thus insulin resistance and/or relative insulin deficiency can lead to blood glucose elevations. It is now recognized that hepatic glucose production and insulin-augmenting incretin hormones, secreted by the gut in response to intestinal nutrients, also play an important role in fasting and postprandial glucose regulation [5].

There is growing sentiment that identifying and treating those with prediabetes is important. Not only do individuals with prediabetes demonstrate increased incidence of diabetes complications [2], but without intervention, 37% to 70% are expected to develop diabetes within 4 years [3, 6]. The Diabetes Prevention Program [7] and two other large prospective studies in Finnish [8] and Chinese [9] populations show that lifestyle interventions successfully prevent conversion to diabetes by 58% at 4 years and 45% at 20 years. Furthermore, lifestyle intervention per quality-adjusted life year costs a mere $1,100, leading public health experts to conclude that lifestyle changes in individuals with prediabetes are cost effective and should be implemented for diabetes prevention [10]. More accurate and/or earlier identification of individuals at risk for type 2 diabetes would thus represent an important public health goal.

While the cornerstone of lifestyle interventions is weight loss and exercise, specific nutrient components may yield further benefits. Recent studies suggest that the positive outcome of dietary intervention to lower glycemic excursions may be improved by using personalized dietary recommendations. Zeevi and colleagues found that the postmeal glucose response to identical foods varies across individuals [11]. This is an important finding, given that the American Diabetes Association (ADA) dietary recommendations are based mainly on reduction of carbohydrate content, without consideration for interpersonal variation in dietary response [1]. The authors were able to build a machine learning model based on postmeal glucose concentrations from continuous glucose monitoring (CGM) data and standardized meals that could accurately identify certain foods as "good" or "bad" for an individual's glucose response [11]. They further showed that generating personalized recommendations based on this model improved postmeal glucose responses, showing the efficacy of personalized nutrition recommendations in controlling glucose dysregulation.

Current diagnostics for both diabetes and prediabetes rely on either HbA1c or single-time-point measurements of blood glucose concentration; as such, they cannot describe the nuances of glycemic patterns. Accumulating evidence suggests that not only do these measures miss a substantial portion of glucose elevations into the prediabetic and diabetic range [12] but also that glycemic variability, or postprandial glucose peaks, may be a metric of even greater importance than current measures of hyperglycemia such as HbA1c or single-time-point measurements when it comes to predicting cardiovascular risk [13–15]. Indeed, postprandial hyperglycemia induces oxidative stress, hypercoagulability, endothelial dysfunction, and inflammation [16–19]. These studies indicate that identification of glycemic variability should be an important consideration in risk stratification and possibly in guiding interventions, including diet, designed to minimize glycemic variability [20].

A comprehensive metric of glycemic variability should encompass not only the magnitude of fluctuations but also the rate of change, relative glucose concentration, and frequency of fluctuations. While current metrics of glucose variability measure individual aspects of the time-series data, none measure the entire temporal profiles. Technological advances in both wearable devices and time-series data analysis enable the characterization of glucose variability by using the shape of continuous blood glucose curve. By analyzing the shape of the continuous glucose time-series data, all aspects of variability can be simultaneously compared. Spectral clustering and new distance metrics have already been used to classify time-series patterns in other disciplines. For example, temporal patterns of energy consumption have been used to predict consumer electricity consumption and to classify patterns of heart rhythm disturbances from electrocardiograms [21, 22].

In this work, we propose the development of a new measure of glucose variability derived from the spectral clustering of glycemic signatures using time-series-specific distance metrics. We show that this method can be used to define a clinically relevant metric of glycemic patterns that would classify individuals into different glucotypes. A summary metric of glucose variability that encompasses all components of glucose signatures is expected to provide a more comprehensive, dynamic, and granular understanding of diabetes etiology, detect glucose dysregulation at earlier stages of disease, and provide a tool by which one can personalize diet for optimal glucose response. We find that many individuals not known to be prediabetic by standard measures (fasting glucose, oral glucose tolerance test [OGTT], and HbA1c) have high levels of postprandial glucose, similar to those characteristic of prediabetes and even diabetes, and can be identified by our approach. Although postprandial elevations in glucose into the diabetic range have been reported previously in up to 9% of normoglycemic individuals, the prior studies primarily relied on static measures of HbA1c and fasting glucose to define normoglycemia and did not use standardized meals to follow postprandial glucose elevation [12, 23, 24].

We designed a study to characterize detailed glycemic patterns both in natural environments and using standardized meals to follow glucose dysregulation using CGM, and used pattern analysis to define individual glucotypes. Using our detailed clinical and CGM measurements, we developed models for glucose dysregulation at an individual level and related these patterns to standard measures of glucose intolerance and diabetes as well as clinical metabolic measures, including whole-body insulin resistance and insulin secretion.

## Results

### Cohort characteristics

**Fig 1:**
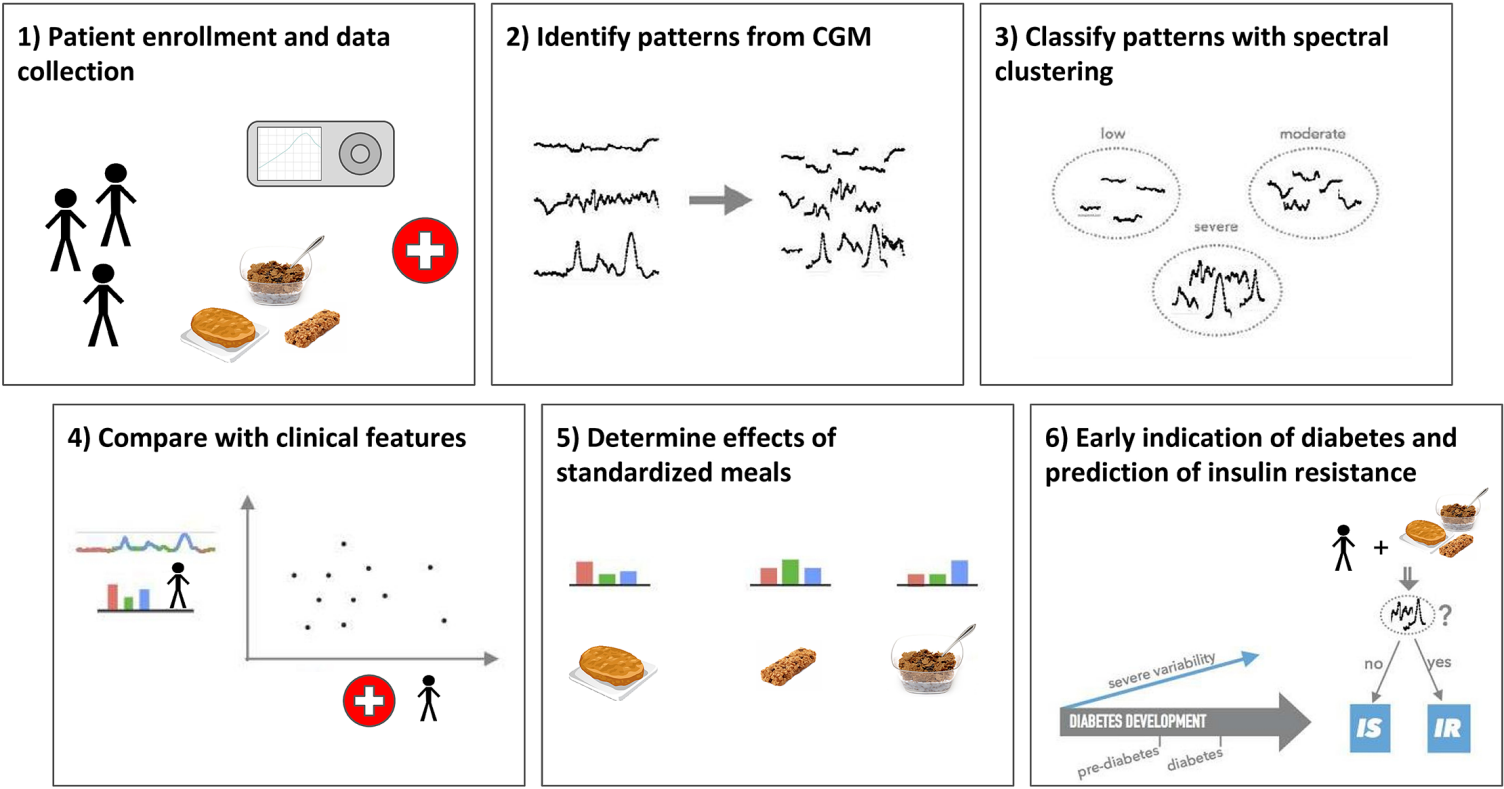
Summary of methods. We enrolled 57 participants with different diagnoses for diabetes and continuously monitored their interstitial glucose levels for 2–4 weeks. Spectral clustering was used to classify different patterns of glycemic responses based on their variability. We then compared different classes of glucose variability with common clinical parameters and in relation to the effect of meal with standardized nutrient content. Finally, we analyzed insulin metabolism to elucidate potential physiological mechanisms underlying glycemic dysregulation detected through our classification. CGM, continuous glucose monitoring; IR, insulin resistance; IS, insulin sensitivity.

We recruited 57 healthy participants without prior diagnosis of diabetes. We monitored their blood glucose using CGM in their normal environment, and we extensively characterized them with clinical metabolic phenotypes including whole-body insulin resistance and insulin secretion (see Fig 1 and Methods for an overview of the study design). The cohort composition was 32 females and 25 males, with an age range of 25 to 76 (median 51). On screening tests, 5 met criteria for having type 2 diabetes, defined as HbA1c ≥ 6.5%, fasting blood glucose ≥ 126 mg/dL, or 2-hour glucose during 75 gram OGTT ≥ 200 mg/dL; 14 had prediabetes, defined as HbA1c > 5.7% and < 6.5%, fasting blood glucose 100–125 mg/dL, or 2-hour glucose during OGTT 140–199 mg/dL; the remainder were normoglycemic, defined as fasting and 2-hour OGTT plasma glucose and HbA1c below the diagnostic thresholds for prediabetes and diabetes (see S1 and S2 Tables for details on the cohort). Mean fasting glucose was 93 mg/dL, 2-hour glucose 125 mg/dL, and HbA1c 5.4%. Insulin resistance, quantified by the steady-state plasma glucose (SSPG) test, in which a higher value indicates relative resistance to insulin-mediated glucose uptake, ranged from 45 mg/dL to 335 mg/dL, reflecting great heterogeneity in the cohort. This measure was particularly variable among the normoglycemic and prediabetic group. Thirty subjects completed the standardized meal testing portion of the study. Characteristics of this cohort mirrored the original cohort: 20 females and 10 males, with age ranging from 25 to 65, of which 3 and 7 individuals were diagnosed with diabetes and prediabetes, respectively.

### Defining glucose fluctuation patterns

**Fig 2:**
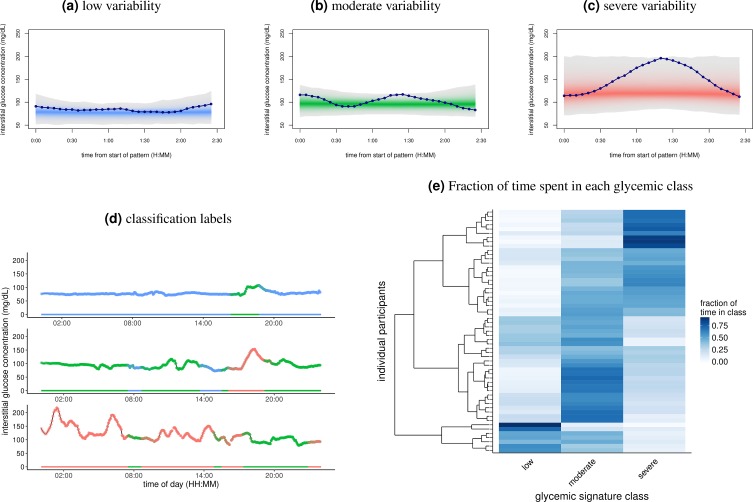
Classification of CGM with classes of glycemic signatures. (A-C) Segregation of the 2.5-hour windows into the three classes of glycemic signatures derived from spectral clustering. The lines in each panel show an example of the glycemic signatures in each class. This separation of windows explains approximately 73% of the variance. (D) One day of CGM data for 3 separate individuals. Color indicates classification of glycemic signatures. Note that since overlapping windows were used for clustering and classification, some periods of the day have multiple classifications. (E) Heat map showing the fraction of time individuals spent in each of the glycemic classes. Rows represent unique individuals in the cohort, while columns represent each of the glycemic signature classes shown in A-C. Color of the tiles corresponds to the fraction of time spent in each class, with 1 being 100% of the time. There were 238 windows per participant (S3 Data). Rows of individuals are arranged according to hierarchical clustering. CGM, continuous glucose monitoring.

Visual inspection of CGM glucose levels of 57 participants wearing continuous glucose monitors revealed highly variable intra- and interpersonal patterns of fluctuation (Fig 2, S1 Data). In an attempt to systematically capture and classify the magnitude and degree of glucose variability, we clustered the temporal profiles of blood glucose responses of the 57 participants, testing a variety of approaches, parameters, and distance metrics (see Methods and S1 Fig, S2 Data). The temporal profiles were segmented into sliding windows of 2.5 hours, with a 75% overlap. Complexity-invariant dynamic time warping (CID-DTW) was used to compute the dissimilarity matrix between each pair of windows. By applying spectral clustering on this dissimilarity matrix, we defined 3 clusters of glucose patterns (S3 Data). Dynamic time warping (DTW) to compute distances between time windows exploits the temporal nature of the data and captures high similarities between windows with similar profiles, even when they are shifted. As such, this method was successful in capturing 73% of the variance, and silhouette analysis indicated a good fit for most of the windows to the groups identified by the spectral clustering (S2 Fig, S4 Data).

Visual inspection revealed that the three types of patterns captured features beyond absolute glucose value, including overall variability and the dynamics of increase and decrease in slope (Fig 2A–2D). Using the spectral clustering method, based on the amount of variability exhibited in glucose levels, we could classify the three patterns as low, moderate, and severe variability. These groups show a progressive increase in both the severity and magnitude of the variability in glucose concentration. We determined the fraction of time each participant spent in each pattern and found that some participants stayed predominantly in the low variability range, whereas others were predominantly in the moderate and severe variability range (Fig 2E), with other clear intermediates as well.

We next assigned the individuals to the “glucotype” associated to their most frequent variability pattern—low variability (glucotype L), moderate variability (glucotype M), and severe variability (glucotype S)—based on the frequency of occurrence in the low, moderate, and severe glycemic signatures (see Methods). This classification of glycemic signatures is consistent with differences in common metrics of glycemic variability based on an ANOVA analysis (S3 Table). In addition, the mean variability values for these metrics increased from low, moderate, to severe glucotype, supporting the naming scheme for the classes indicating increasing variability. Note that the classes increase in both variability and mean glucose concentration (77, 96, and 122 mg/dL in low, moderate, and severe classes, respectively), making them a more comprehensive metric of glycemic state.

### Correlation with clinical and metabolic parameters

**Fig 3:**
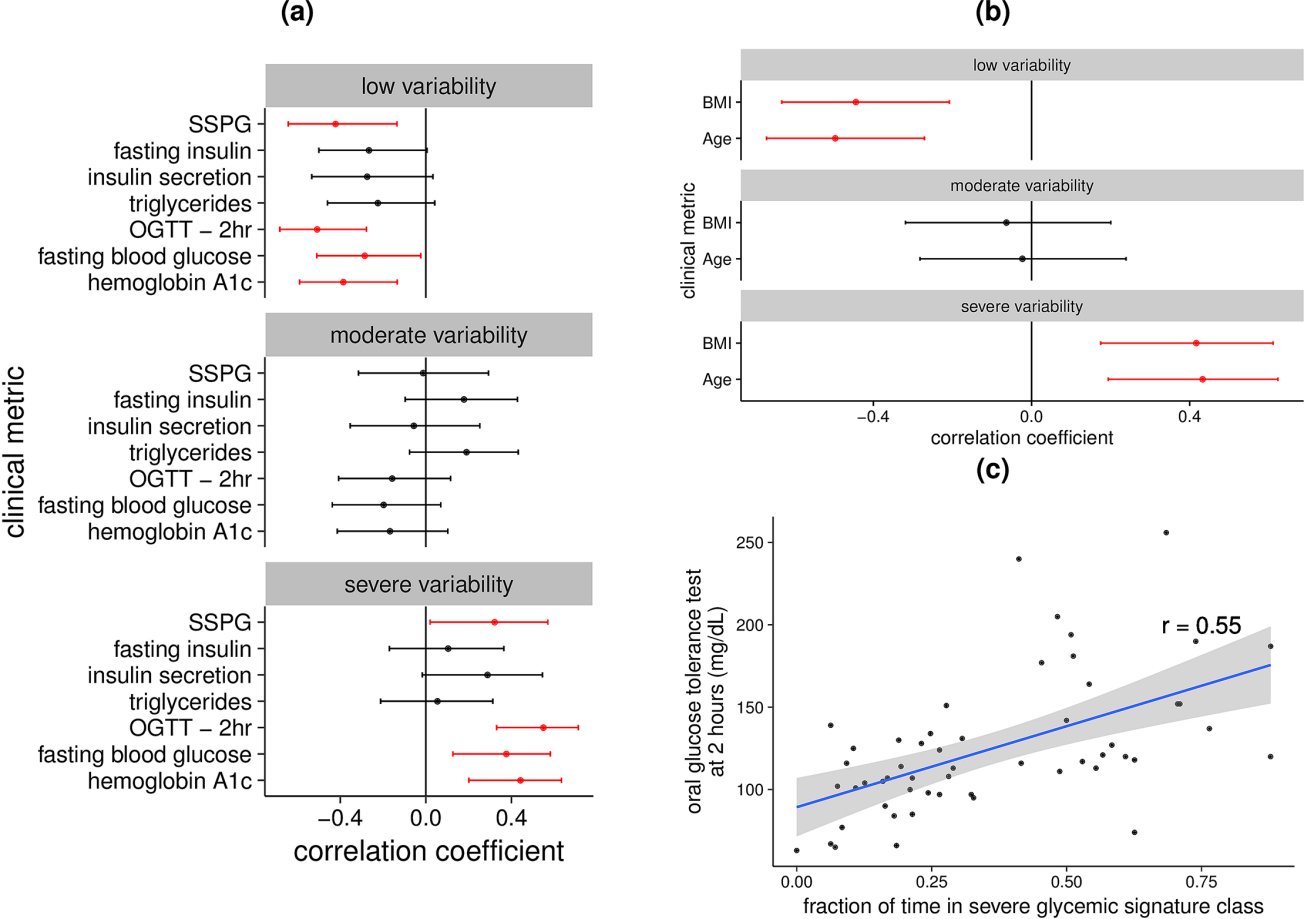
Correlation between glycemic signature classes and measures of glucose homeostasis. (A) Forest plots for each of the glucotypes. A Pearson’s correlation test was used to determine the correlation between the clinical metabolic tests—listed 1 per line—and the fraction of time spent in each glucotype class (S5 Data). The center dot line is the resulting correlation coefficient, with the line representing the corresponding 95% confidence interval. (B) Forest plot with the lines representing age and BMI. (C) OGTT 2hr is plotted against the fraction of time in the severe glucotype for each individual. The line of best fit is shown in blue with the 95% confidence interval shaded in gray. The correlation coefficient, r, was derived from a Pearson’s correlation test. OGTT 2hr, blood glucose concentration 2 hours after the start of oral glucose tolerance test; SSPG, steady-state plasma glucose.

We next correlated the fraction of time spent in each glucotype pattern with clinically relevant metabolic measures (S3 Fig, S4 Table, S5 Data). We found significant positive correlations (*P*<0.05) between the time spent in low glycemic signatures and lower values for fasting glucose, HbA1c, OGTT, SSPG, BMI, and age (Fig 3). Similarly, the frequency of time spent in severe glycemic signatures was associated with higher values for fasting glucose, HbA1c, OGTT (Fig 3C) and SSPG, BMI, and age (Fig 3).

### Glucose patterns and standardized meals

To better assess glucose variability among individuals, we next subjected a subset of the participants (30) to 3 standardized meals, which contain similar calories but vary in their amounts of proteins, fat, and fiber. Meals were consumed at breakfast, which is when participants had a stable baseline. The meals were cornflakes and milk (low fiber and high sugar), a peanut butter sandwich (higher fat and higher protein), and a PROBAR protein bar (moderate fat and protein) (S5 Table). Each participant received the meal twice, and for each participant, good reproducibility was observed between replicates (0.5 and 0.4 average Pearson’s correlation coefficients between replicates and between individuals, respectively, *p*-value = 1e – 08, wilcoxon test).

**Fig 4:**
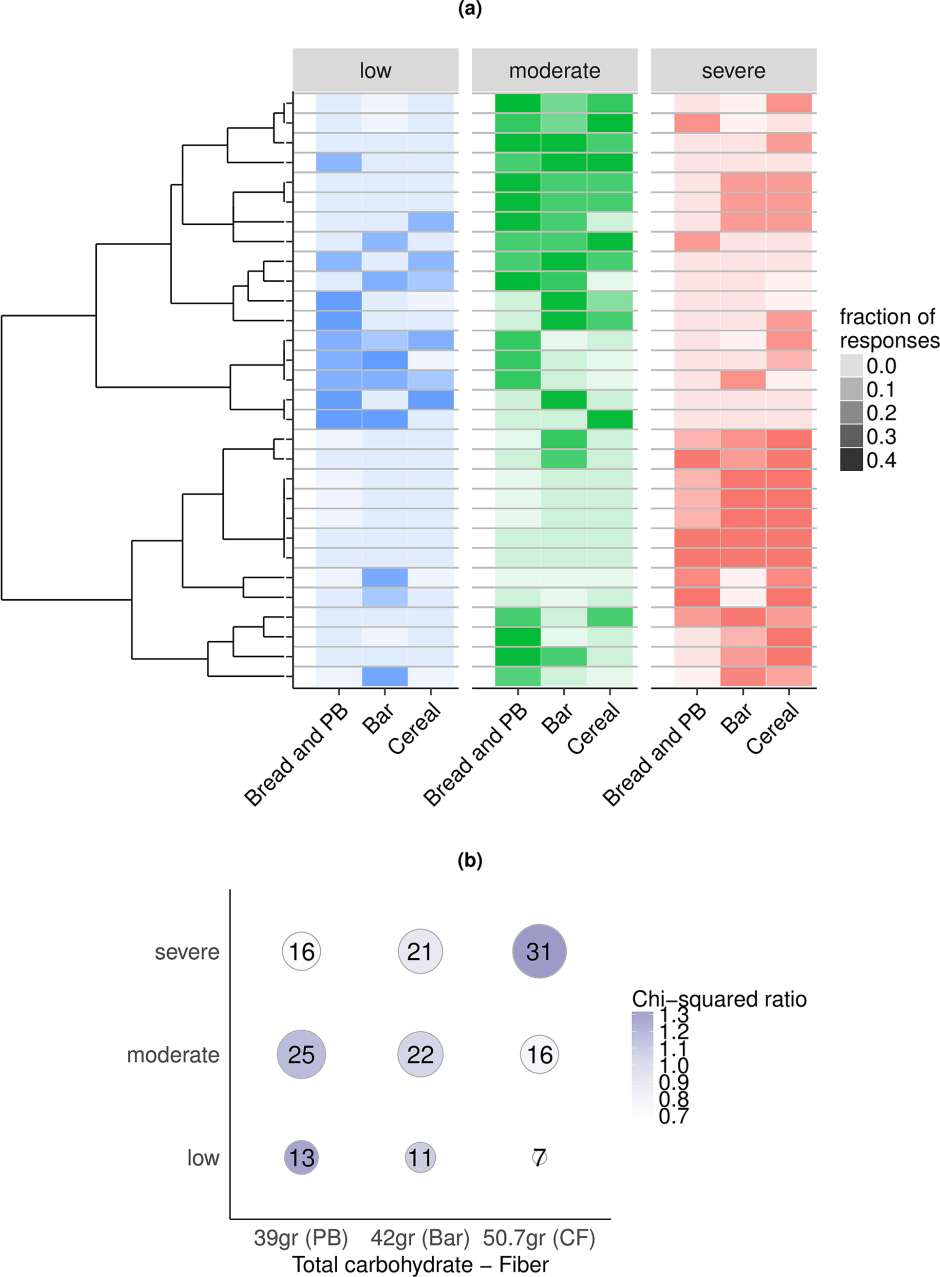
Correlation between carbohydrate content and frequency of severe glycemic signature responses. (A) Heat map of glycemic signature class responses to standardized meals. Rows represent individuals, and columns represent meals. Color indicates classification of response, and intensity indicates fraction of total responses (S7 Data). The number of total responses per individual corresponds to the number of standard meals each participant ate. Individuals are sorted by hierarchical clustering (left) based on the fraction of responses in a given class, regardless of the type of meal that triggered the response. (B) Contingency table reporting the number of responses to standardized meals assigned to a given glycemic signature class. Meals are sorted based on their net carbohydrate content (total carbohydrates–fiber). The size of the dots is proportional to the number of windows, and the intensity is the chi-squared ratio between observed and expected counts. There is a significant association between net carbohydrate content and severity of the response (*p*-value: 0.06, chi-squared test). “PB”: bread and peanut butter; “Bar”: PROBAR protein bar; “CF”: cornflakes and milk. For both panels A and B, the classification of glycemic responses to meals is based on the entire CGM profiles rather than on the initial set of clustered windows (see Methods). CGM, continuous glucose monitoring.

Several types of meal responses were found in the cohort (Fig 4A, S4 Fig, S6 Data, and S7 Data, see Methods for details on the classification of meal responses). The vast majority (60%) of the responses to milk/cereal were classified as severe variability, whereas the responses to the PROBAR protein bar and to bread and peanut butter varied among individuals. Importantly, 16 subjects who were classified as “normal” based on current clinical tests for diagnosing diabetes had glucose levels in the prediabetic (>140 mg/dL) or diabetic (>200 mg/dL) range after the consumption of 1 or more of the standardized meals (S4 Fig). Additionally, 25 subjects had higher glycemic responses measured by CGM following mixed meals than the responses noted on the OGTT, even with similar carbohydrate loading.

To determine the effect of nutrition on these responses, we correlated the number of low, moderate, and severe responses with the nutritional content of the meals (Fig 4B). As expected, lower fiber content in cornflakes and milk is associated to more severe responses (Fig 4B, *p*-value = 0.06, chi-squared test). On the other hand, bread and peanut butter, which has more fiber, more fat, and more protein, elicits more low or moderate responses (Fig 4B).

### Diabetes classification and variability frequency

**Fig 5:**
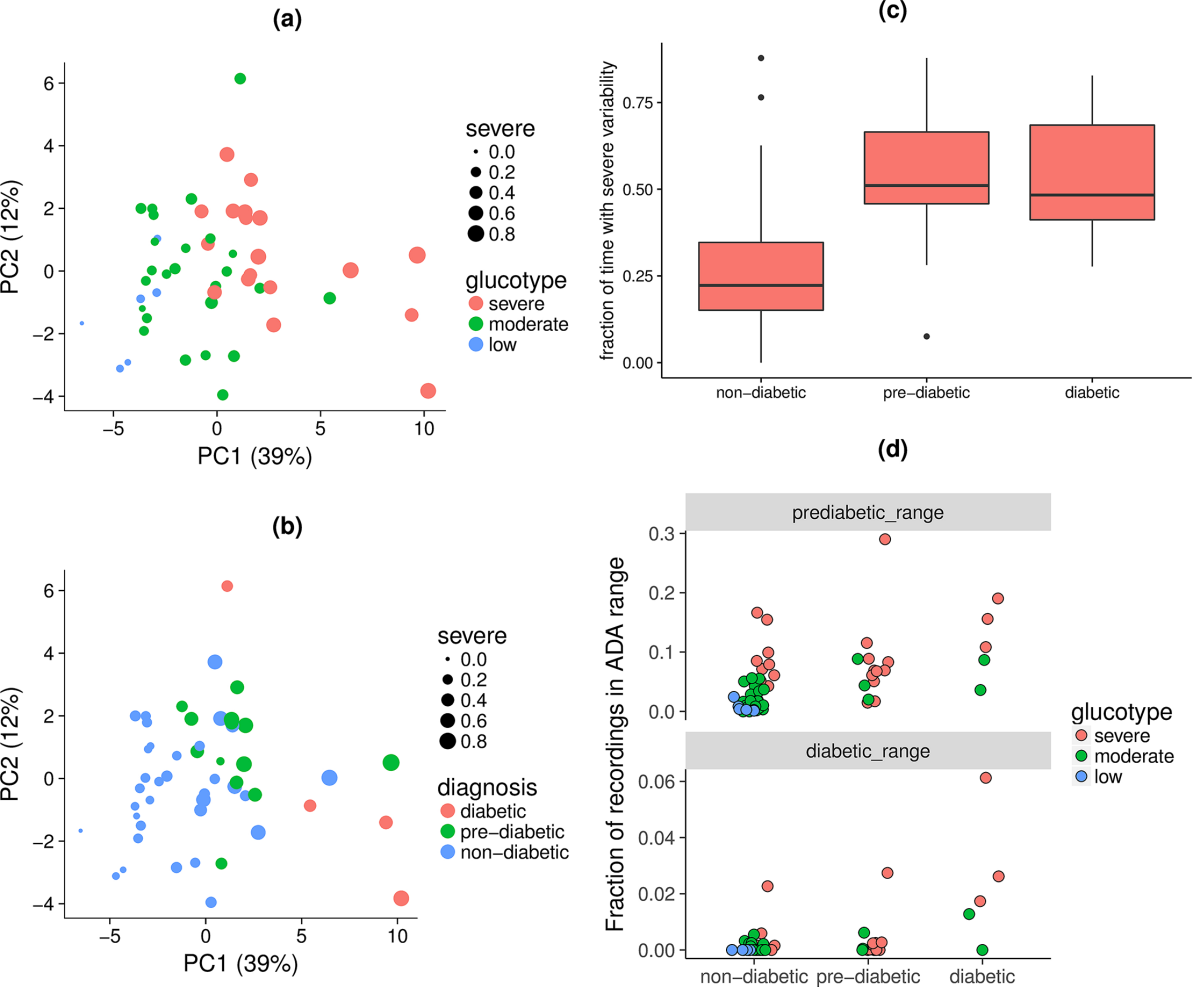
Diabetes classification and prevalence of severe variability. (A) Principal component (labeled “PC”) analysis of common measures used to describe glucose control and evaluate CGM data (S5 Data). Individual participants are colored based on their glucotype, the glycemic signature class in which they spent the majority of their time. The dot size is proportional to the fraction of time spent with severe variability. (B) Same principle component analysis, but participants are colored based on diabetes diagnosis. This diagnosis was based on the ADA Guidelines of HbA1c, fasting blood glucose, and blood glucose concentration at 2 hours after the start of an OGTT. (C) Box and whisker plot of fraction of time spent with severe variability for nondiabetic, prediabetic, and diabetic individuals. The classification is based on all time windows, not restricting to the ones after standardized meals. (D) Proportion of CGM data in prediabetic and diabetic glycemic ranges defined in the ADA Guidelines. Participants are grouped by their diabetes diagnosis and colored by their glucotype. Some normoglycemic participants who demonstrated the severe glucotype reached prediabetic glycemic levels up to 15% of the time and diabetic glycemic levels 2% of the time. ADA, American Diabetes Association; CGM, continuous glucose monitoring; OGTT, oral glucose tolerance test.

In order to determine the clinical relevance of these glucotypes in relation to diabetes classification, we examined the frequency of these classes in comparison with diabetes diagnosis using the OGTT results. A principal component analysis was then used to separate participants by their ability to maintain glucose homeostasis as assessed by clinical glucose metabolic phenotypes and CGM metrics, such as mean amplitude of glycemic response. The first two components explained slightly over half of the variation in blood sugar control (51%) and are shown in Fig 5A and 5B. Glucose control decreases along both principal component one and principal component two, such that the nondiabetic participants are located in the lower-left corner. Many of those with prediabetes were already dominated by severely variable glycemic signatures, which would be expected of diabetic individuals (Fig 5A, 5B and 5C). Furthermore, we observed that even participants clinically undiagnosed with diabetes or prediabetes can have glucose spikes in prediabetic or diabetic range according to the ADA thresholds (Fig 5D). Indeed, normoglycemic patients classified as severe glucotype (24% of normoglycemic) can reach prediabetic glucose levels up to 15% of the duration of CGM recordings and diabetic glucose levels during 2% of the recordings (Fig 5D). Thus, normoglycemic individuals can exhibit severe glucotypes with postprandial response similar or exceeding those of diabetics.

### Individualized differences for glucose dysregulation

**Fig 6:**
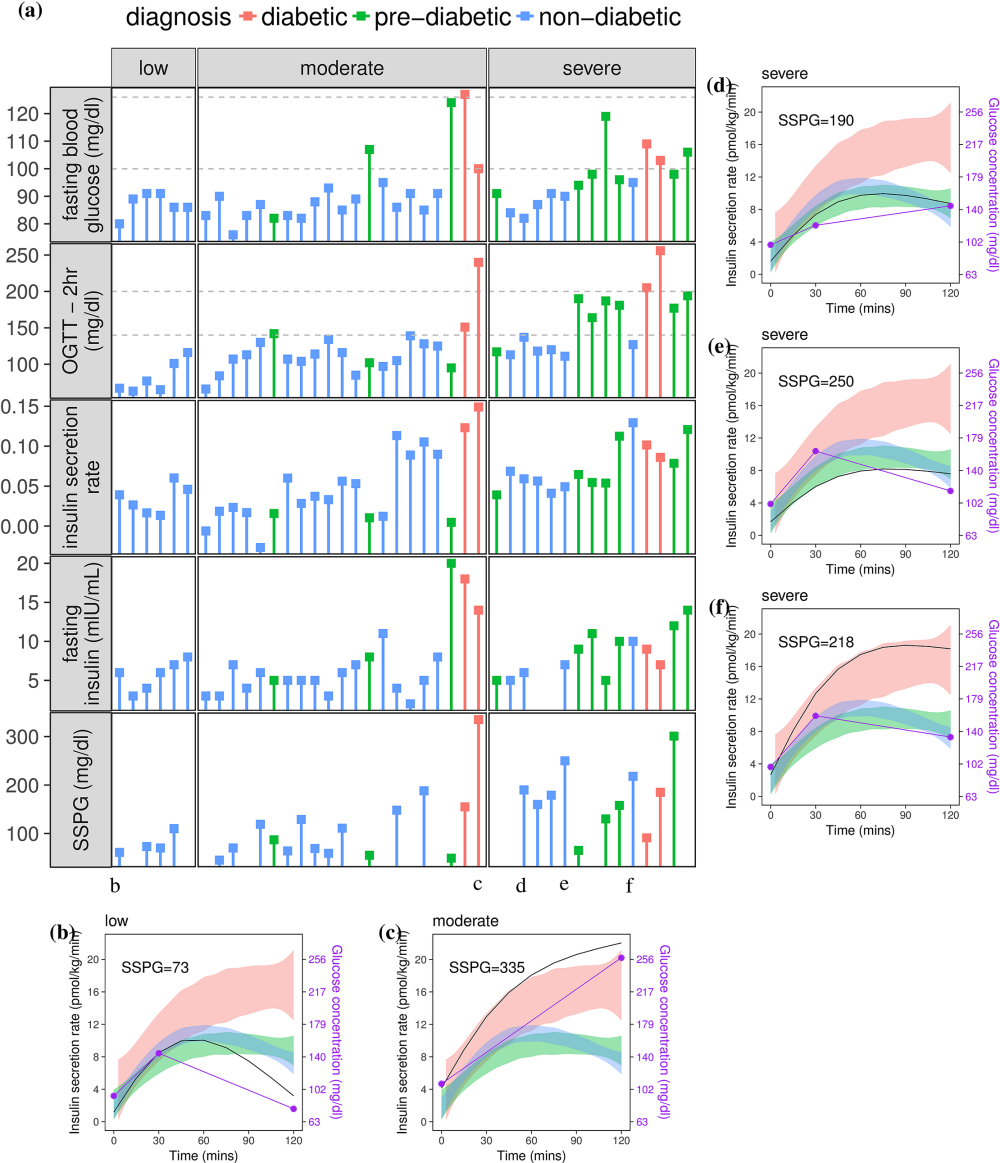
Glucotypes and insulin metabolism. (A) Comparison of glycemic response, insulin secretion, and insulin sensitivity across different glucotypes (low, moderate, severe) (S5 Data). Each column is a participant. The glycemic response is shown in the horizontal panels as fasting blood sugar concentration and as blood glucose concentration 2 hours after oral glucose load in an OGTT. Dashed lines for fasting blood sugar and 2-hour glucose in OGTT correspond to the ADA thresholds for prediabetes and diabetes. Insulin secretion rate was calculated using deconvolution of C-peptide concentrations at 0, 30, and 120 minutes during OGTT (ISEC software [25], see Methods). The average derivative of the insulin secretion curve is shown as aggregate measure of insulin secretion rate over the whole OGTT time course. Insulin sensitivity is shown as glucose concentration from SSPG test, with higher values reflecting greater insulin resistance. (B, C, D, E, F) Insulin secretion rate (black solid line) and blood glucose concentration (dots and purple solid line) during OGTT for 5 individuals (S8 Data). Shaded areas in panels B-F aggregate data of distribution of insulin secretion rate for participants diagnosed with diabetes (pink), prediabetes (green), and nondiabetic (blue). Each panel represents an individual with a different physiology. (B) Insulin-sensitive individual with normal insulin secretion and a low blood glucose 2 hours postprandially; (C) Individual diagnosed with diabetes demonstrating high glycemic concentrations despite high insulin secretion; (D) Nondiabetic individual with normal fasting blood glucose but high 2-hour OGTT value in setting of insulin resistance and absolute but relative deficiency of insulin secretion; (E) Nondiabetic individual with insulin resistance with low insulin secretion, characterized by early glucose rise after load; (F) Nondiabetic individual with insulin resistance and high compensatory insulin secretion with relatively normal postprandial glucose following oral glucose load. ADA, American Diabetes Association; ISEC, Insulin SECretion; OGTT, oral glucose tolerance test; SSPG, steady-state plasma glucose.

There are a number of possible physiological reasons for increased glucose variability, including, but not limited to, insulin resistance and impaired insulin secretion. To further examine the factors underlying variable glucose patterns described by glucotypes, we compared insulin secretion rate, insulin resistance, and insulin concentrations with regard to glucotype patterns (see Methods and Fig 6A, S8 Data). To demonstrate heterogeneity in insulin metabolism relative to insulin resistance and glucose profiles, dynamic metabolic responses for several individuals are shown in Fig 6. In general, patients diagnosed with diabetes showed sustained insulin secretion in response to the oral glucose load, although their insulin sensitivity and glycemic responses varied. As an example of normoglycemic participants with low glucose variability, Fig 6B shows an insulin-sensitive individual with normal insulin secretion and a low blood glucose 2 hours postprandially. In contrast, Fig 6C represents an individual diagnosed with diabetes demonstrating high glycemic concentrations despite high insulin secretion. Since they have elevated SSPG levels, this individual is deficient in glucose uptake (i.e., insulin resistant). We then examined insulin metabolism and glucose response in 3 undiagnosed individuals according to standard clinical parameters (Fig 6D–6F). These include a nondiabetic individual with normal fasting blood glucose but high 2-hour OGTT value in setting of insulin resistance and relative deficiency of insulin secretion (Fig 6D); a nondiabetic individual with insulin resistance and low insulin secretion, characterized by early glucose rise after load (Fig 6E); and a nondiabetic individual with insulin resistance and high compensatory insulin secretion with relatively normal postprandial glucose following oral glucose load (Fig 6F). Other combinations also exist. Thus, different individuals have distinct mechanisms that are likely responsible for glucose dysregulation.

### Web interface for glucotype classification

To demonstrate the applicability of our classification algorithm, we designed a single-page web interface that enables the visualization of a CGM profile as well as the classification into glucotypes. The CGM viewer is implemented in R as a shiny app [26] and is available at glucotype.stanford.edu. Although still in a prototyping state, it allows the user to upload 2–3 weeks of CGM recordings as a tab-delimited file and interactively displays the glucose values along time. At the user's request, the profile is fragmented into sliding windows of 2.5 hours, which are colored based on their glycemic signature class. In addition, a table shows the fraction of time in each category, for which the most frequent category is indicative of the user's glucotype.

## Discussion

In this study, we recorded almost 500,000 measurements from 57 participants. We observed a considerable amount of intra- and interpersonal variability in glucose measurements. Spectral clustering revealed 3 glucotypes of increasing variability (low, moderate, and severe)—each also characterized by increasing mean glucose—that explained 73% of the observed variance. The fraction of time spent in low and severe variability patterns correlated with standard measures of glycemia associated with diabetes risk. However, within traditional classification categories based on fasting and 2-hour glucose (OGTT) or HbA1c, all three glucotype patterns could be observed, indicating that current classification schemes are overly simplistic. Of great interest is the possibility that identification of those with severe glycemic variability within the group of normoglycemic or prediabetic individuals would enhance prediction of risk of progression to diabetes. Indeed, severe glucose variability was present in 25% of normoglycemic individuals, and within this subgroup, glucose reached prediabetic or diabetic glucose levels 15% and 2% of the time, respectively. We speculate that an increase in glucose variability might thus precede the currently used measures defining abnormal glucose levels and thus represent an even earlier stage of “prediabetes.” Longer-term studies are warranted to define whether identification of a “severe glucotype” via CGM has greater predictive value for development of type 2 diabetes than do traditional tests. The fact that CGM obtains glucose excursions in a real-living situation as compared to the artificial glucose tolerance test may be of added value in this regard.

Recent evidence suggests that glycemic variability, more than fasting glycemia or HbA1c, predicts development of cardiovascular disease, possibly via oxidative damage causing endothelial dysfunction [16–19]. We describe a classification of glycemic signatures that can encompass not only the magnitude of fluctuations but also the rate of change, relative glucose concentration, and frequency of fluctuations. This classification splits glycemic signatures into 3 glucotypes, which represent increasing levels of variability according to several of the most common measures of CGM variability used in the literature. Such a categorization of glycemic signatures that considers multiple aspects of “variability” may serve as a more comprehensive measure of glucose variability. As continuous glucose monitors become more accessible and affordable with newer technologies on the horizon, using this metric of glycemic variability may enable earlier and possibly expanded detection of individuals at risk for type 2 diabetes and cardiovascular disease. If validated by long-term studies, use of CGM to define glucotypes might become an important tool for clinicians, and even individuals outside the medical system, to stratify their risk and to adopt interventions shown to prevent diabetes and cardiovascular disease.

A second limitation of traditional methods for measuring glucose dysregulation is that they do not reveal the complexity of glycemic patterns that differ between individuals and that may reflect underlying physiology (e.g., insulin resistance versus B cell dysfunction/insulin secretion) and/or risk of progression to type 2 diabetes. Glucotype patterns obtained from CGM have potential to define the dominant underlying physiologic basis for glucose dysregulation for a given individual. The deterioration of glucose metabolic control is determined by a variety of mechanisms that include peripheral insulin resistance (muscle and adipose tissue), hepatic insulin resistance, insulin secretion, and the incretin effect on insulin response. Most likely, a complex interplay between all of these metabolic processes determines the glycemic patterns, and, at an early stage, while fasting and/or 2-hour glucose on OGTT may still appear normal, glycemic variability as detected on CGM could reveal early abnormalities as well as dominant physiologic basis (e.g., muscle versus beta cell versus liver versus incretin response). Although we did not quantify incretin response or hepatic insulin resistance in this study, the current results highlight how use of glucotype can identify the underlying physiology in subgroups of those with ADA-defined prediabetes or normoglycemia.

It is interesting to note that CGM severity correlates with OGTT, but they are not identical, and a number of individuals with normal OGTT responses exhibited severe glucose variability. In a different study, Maaze and colleagues reported similar results [23]. The difference in OGTT measurements, which relies on pure glucose, as compared with CGM may be due to several possibilities. First, current measures are limited by the OGTT measure itself, which relies on two glucose values obtained on a single day, which fails to capture sufficient data to identify individuals at early states of glucose dysregulation. Second, it is possible that specific foods, amounts, and mechanisms of glucose delivery and removal to individuals cause greater glucose elevation in natural setting than delivery of pure glucose. These possibilities are not mutually exclusive. Regardless, our proposed glycemic variability metric has the advantage of classifying individuals into risk categories more accurately and in a more natural environment than current clinical tests. Having individuals use a wearable device to examine glucose response outside of the clinic in their normal environment provided more granular information than the snapshot of clinical phenotypes typically used. Importantly, 9 out of the 23 individuals classified as glucotypes S would not be diagnosed with prediabetes or diabetes using the traditional diagnostic tests of fasting blood sugars, 2-hour OGTT, or HbA1C. These individuals could start lifestyle modifications that would reduce their risk of developing disease at a much earlier stage. For example, the three individuals in Fig 6D, 6E and 6F had a BMI in the overweight range, suggesting that glucotype classification can help identify subjects with altered glucose homeostasis resulting from diverse metabolic phenotypes, for which weight loss interventions might be beneficial. Based on our results with the standardized meals, these individuals would be able to lower the glucose variability by decreasing carbohydrate load and choosing more complex carbohydrates and including protein in their meals. Importantly, since different people respond differently to the same meals (a result noted by Zeevi and colleagues [11]), it should be possible to personalize dietary programs and thereby manage glucose elevations at an individual level; CGM will be valuable for this purpose.

It is interesting to note that although individuals respond differently to different foods, there are some foods that result in elevated glucose in the majority of adults. A standardized meal of cornflakes and milk caused glucose elevation in the prediabetic range (>140 mg/dl) in 80% of individuals in our study. It is plausible that these commonly eaten foods might be adverse for the health of the majority of adults in the world population.

In summary, this study describes the development of a new glucose variability metric derived from the use of a wearable CGM device. With this metric, we categorized individuals into one of three glucotypes—low (L), moderate (M), or severe (S)—which allowed us to accurately identify individuals with aberrant glucose metabolism as measured by standard tests and to further identify aberrant glucose metabolism in those who would have appeared normal by standard tests. Thus, we found that some individuals with normoglycemia by OGTT already have glucose dysregulation, potentially due to insulin resistance and relative insulin secretion defects. The factors underlying the physiologic basis for glycemic variability appears to be heterogeneous and merits further characterization in relationship to specific glucotype patterns. Further, we demonstrated heterogeneity in glycemic responses to standardized meals, even within traditional ADA-defined glucose-tolerant categories. Thus, the results of the current study highlight individual variability in glycemic responses to oral nutrients and the ability to detect clinically significant degrees of hyperglycemia that would not have been identified using traditional tests. The potential for early risk detection with use of CGM and, in particular, the predictive value of identifying “severe glucotype” need validation by long-term follow-up studies. As glucose monitors become more accurate, less invasive, and less expensive, their use will increase both in the diabetic, prediabetic, and healthy population. A glucotype classification system such as the one described here would assist in identifying those individuals with high glucose variability, and future long-term studies are needed to confirm if these individuals are at increased risk for developing diabetes and cardiovascular disease. Furthermore, such a system may help pinpoint the relatively dominant physiologic defect underlying dysglycemia, which in turn may point to specific interventions/diet to address abnormal glycemic patterns.

## Methods

### Ethics statement

The study was approved under IRB 37141, and written consent was obtained for all participants.

### Subjects and overall design

Human subjects were recruited from the San Francisco Bay Area via local newspaper advertisements and informational lectures to the community. All subjects provided written, informed consent, and the protocol was approved by the Stanford Internal Review Board. Subjects were required to be healthy and free of major organ disease, chronic inflammatory conditions, malignancy, uncontrolled hypertension, eating disorder, history of bariatric surgery, diagnosis of diabetes, use of weight loss or diabetogenic medications, or recent unstable weight. Screening and eligibility determination were conducted in the Stanford Clinical Translational Research Unit (CTRU) with history and physical exam, fasting plasma glucose, and HbA1c. All subjects underwent home blood glucose monitoring using CGM in their usual environment to identify patterns of glucose variability (Fig 1). They then underwent extensive metabolic phenotyping including fasting blood glucose, OGTT with fasting and postchallenge insulin concentrations, HbA1C, and quantitative insulin-mediated glucose uptake testing via the SSPG test (see Metabolic phenotyping section). On screening tests, 5 met criteria for having type 2 diabetes, defined as HbA1c ≥ 6.5%, fasting blood glucose ≥ 126 mg/dL, or 2-hour glucose during 75 gram OGTT ≥ 200 mg/dL; 14 had prediabetes, defined as HbA1c > 5.7% and < 6.5%, fasting blood glucose 100–125 mg/dL, or 2-hour glucose during OGTT 140–199 mg/dL; the remainder were normoglycemic, defined as fasting and 2-hour OGTT plasma glucose and HbA1c below the diagnostic thresholds for prediabetes and diabetes. Three standardized meals prepared by study dietitian, with varied carbohydrate, protein, and fat content, were administered for characterization of the variability of glycemic response as measured by CGM. Responses to these meals and to home CGM monitoring were scored and analyzed relative to diabetes diagnosis and other clinical glucose metabolic patterns.

#### Continuous blood glucose monitoring

Dexcom G4 CGM devices, which provide interstitial glucose concentrations every 5 minutes, were placed on participants in the Stanford CTRU and worn by participants for a minimum of 2 weeks and maximum of 4 weeks during a period of stability in their lifestyle (no vacations, holidays, festivities, etc.). Participants were instructed to calibrate monitors once to twice daily using glucose meters (AccuCheck Nano SmartView). Subjects were blinded to the results of monitoring until after the monitors were removed so that their dietary habits were not influenced by the glucose recordings.

#### Metabolic phenotyping

Glucose tolerance was assessed after an overnight fast via OGTT with plasma samples drawn for measurement of glucose (oximetric method) and insulin (radioimmunoassay) at baseline, 30 minutes, and 120 minutes after administration of 75 grams of glucose. From the baseline sample, HbA1c, triglyceride, and high-density lipoprotein cholesterol levels were also determined. Insulin-mediated glucose uptake was quantified via the SSPG test as previously described and validated [27, 28]. Briefly, following an overnight fast, subjects were infused for 180 minutes with octreotide (0.27 *μ*g/m^2^ minutes), insulin (25 mU/m^2^ minutes), and glucose (240 mg/m^2^ minutes). Venous blood was drawn every 10 minutes from 150 to 180 minutes of the infusion: mean glucose and insulin from plasma constitute the steady-state plasma insulin (SSPI) and SSPG concentrations for each individual. SSPI concentrations are similar in all subjects during this test, whereas SSPG concentration provides a direct measure of the ability of insulin to mediate glucose disposal. Individuals that are more insulin resistant would a have higher SSPG concentration. Insulin secretion was calculated from the OGTT as per the section Calculation of insulin secretion rate. The tests were usually performed when the participants were not wearing the Dexcom device.

#### Calculation of insulin secretion rate

Insulin secretion rate was estimated from C-peptide concentration measured during OGTT tests at baseline, 30 minutes, and 120 minutes after administration of 75 grams of glucose. The Insulin SECretion (ISEC) software [25] was used to calculate prehepatic insulin secretion from plasma C-peptide measurements with adjustment for age, sex, and BMI [29]. The ISEC software can be obtained from the author Roman Hovorka, PhD, Metabolic Modelling Group, Centre for Measurement and Information in Medicine, Department of System Science, City University, Northampton Square, London EC1V OHB, United Kingdom. We required an error coefficient of variation of 5% and 15-minute intervals. As the program uses a population model of C-peptide kinetics, we classified individuals as diabetic (“niddm”) if they have either fasting blood sugar > 126 mg/dL, sugar levels after 2 hours during OGTT > 200, or A1C > 6.5; or as”obese” if they are nondiabetic with BMI > 30. The remaining individuals were classified as “normal”.

#### Glycemic responses to standardized meals

A subset of participants were instructed to eat 3 different standardized meals for breakfast: bread and peanut butter; energy bar; or cereal, milk, and raisins (see S5 Table for the nutrient content). While eating these meals, participants wore the Dexcom monitors to record their glucose concentration before, during, and after eating the meal. Participants were instructed to eat each of these meals twice, on 2 separate days, and to record the time of their meals. Several participants neglected to record the time or had additional food with the standardized meals and were excluded from the analysis.

### Classification of glycemic signatures

#### Data preprocessing

Data were extracted directly from the Dexcom G4 CGM system worn by participants. Potential glycemic signatures were generated from overlapping windows. The size of the window and overlap were chosen to optimize clustering performance (S1 Fig, see section below for more details). A window size of 2.5 hours, the approximate time to respond to a meal, was chosen based on both a systematic analysis of its frequency in the power spectrum and its relevance in diabetes. After parameter optimization with a selection of overlaps, the optimal overlap was chosen to be 75%, providing a 4× coverage of each data point.

Linear imputation for gaps under 15 minutes in length was performed in order to reduce the amount of missing data. Windows with larger gaps were excluded from the analysis. Each of these windows was then smoothed using a polynomial smoothing and z-score normalized prior to clustering. The number of windows was then normalized between participants by taking the first *N* windows from the start of the CGM data, in which *N* was the smallest number of windows for a single participant. The clustering is performed on CGM data from all participants at once, including also the ones who did not eat the standardized meals or for whom OGTT data were not available.

#### Parameter optimization

The number of clusters used for parameter optimization is the optimal k from the eigengap heuristic, which corresponds to the distance between consecutive eigenvalues of the spectral clustering. The optimal number of clusters k could vary between different combinations of parameters, and the clustering metrics are computed with respect to a given k for each set of parameters. The optimization of window size and window overlap was based on several clustering metrics (S1 Fig):

- Number of clusters: the optimal number based on the eigengap heuristic
- Proportion of variance explained: total between-cluster sum of square (*totBSS*) divided by total sum of square (*totSS*). The sums of squares were computed on a given distance matrix with the function css() from the R package GMD [30].
- Average silhouette width: the silhouette value is a measure of how similar a window is to its own cluster compared to other clusters. The silhouette index is the average of the silhouette values for all windows. For a window *w*, the silhouette is defined as $s(w)=\frac{b(w)-a(w)}{max(a(w),b(w))}$, in which *b*(*w*) is the lowest average distance of *w* to all points in any other cluster, of which *w* is not a member, and *a*(*w*) is the average distance of *w* with all other windows within the same cluster.
- Calinski-Harabasz (CH) index: ratio between *totBSS* and total within-cluster sum of square (*totWSS*) normalized by the number of windows and number of clusters [31]. Higher CH index means better cluster definition.
- Entropy: similar to the information entropy, it indicates how evenly the windows are assigned to the clusters. Low entropy means unbalanced clusters. Formally, $entropy=-\sum_{i=1}^{k}p_{i}*log(p_{i})$, in which *p*_*i*_ = *size*_*i*_/*W*, *p*_*i*_ > 0, *k* is the number of clusters, and *W* is the total number of windows.
- Dunn index: given a certain distance metric between two clusters, it is defined as the ratio between the minimum pairwise distance over all pairs of clusters and the maximum within-cluster distance (cluster diameter) over all clusters [32]. For a given assignment of clusters, a higher Dunn index indicates better clustering.

The average silhouette, CH index, entropy, and Dunn index were computed with the function cluster.stats() from the R package fpc [33].

The same set of clustering quality metrics was used to select CID-DTW [34] as distance metrics between windows. The other distances tested were euclidean, [35] DTW, and complexity-invariant distance (CID) [34]. CID-DTW was chosen because of both its clustering performance and applicability in comparing glycemic–temporal signatures.

#### Spectral clustering

Spectral clustering was performed according to the methods in von Luxburg's tutorial on spectral clustering [36] on the dissimilarity matrix consisting of pairwise CID-DTW distance between all pairs of windows across all individuals. A symmetric step pattern and Sakoe-Chiba band with a size of 10% the window size were used. The distance matrix was generated using the “DTW” and “Proxy” package in R [35, 37].

The number of neighbors for building the graph for the k-nearest-neighbor affinity matrix (also referred to as the adjacency graph) was optimized by finding the smallest *n* such that the entire graph was connected. In order to optimize this parameter, the affinity matrix and the unnormalized laplacian were calculated for several *n*, and the smallest *n* was chosen such that a single eigenvalue from the unnormalized laplacian was 0.

Clustering was attempted for k– 5 to k + 5 (in which k is the optimal number of clusters from the eigengap heuristic) with the bounds that k must be greater than 2 and less than half the number of windows. An ANOVA analysis was performed to determine the parameters that optimized optimal explained variance and CH indices similar to the analysis done by Bersch and colleagues [38]. The optimal number of clusters (k) was chosen after considering the elbow method, CH index, average silhouette index, and the eigengap heuristic described by Luxburg [36].

#### Classification of new glycemic windows

For most of this study, we analyzed a limited number of temporal windows (238 per person) to ensure the same duration of glucose recordings across all individuals, unless specified otherwise. Nonetheless, spectral clustering can be also used to classify CGM profiles that were not included in the initial clustering. We used this approach to classify all the responses to the standardized meal and to build an interactive web interface in which users can upload their CGM profiles and display their predicted glycemic signatures (see corresponding sections below).

To classify new windows, it is important that the data are processed consistently as the training set. Here, we refer to the initially clustered windows as training set or training windows (238 windows per person). Specifically, the glucose values are mean centered and scaled by using precomputed mean and standard deviation from the training windows. Any new CGM profile has to be fragmented into temporal windows of the same size as the training set (2.5 hours) in order to correctly compute the DTW distance. The overlap between the windows, however, could differ from the training set.

Because of the nature of the algorithm, DTW is a computationally intensive distance metric; therefore, we restrict the computation to only a minimal subset of the training windows. We require a minimum of 200 training windows in total across all patients, and we add more training windows when the number of windows to classify exceeds 200. In the latter case, the number of training windows is the same as the number of windows to classify. To ensure this subset of training windows recapitulates the overall distribution of glycemic variations, we randomly select the training windows—with a fixed random seed—based on their density distribution around the centroids of the three defined classes in the eigenvector space.

CID-DTW is then computed between each pair of new windows and randomly selected training windows. This distance matrix is converted to an affinity matrix following the implementation of the affinityMatrix() function in the Similarity Network Fusion R package (SNFtool [39]). The sigma parameter of the affinityMatrix() function is the precomputed estimate based on the entire training set. The new windows in the affinity matrix are projected onto the eigenvector space, which are derived from the randomly selected windows of the training set, and individually normalized across the eigenvectors. Finally, the windows in the eigenvector space are assigned to a glycemic signature class according to the class of the closest centroid.

To check the reliability of this approach, we classified all the windows in the training set and achieved a remarkable accuracy of 95%. It is important to note that the three classes that we defined—glucotypes—are the results of an unsupervised method. Since we do not have ground truth training labels, we restrain from implementing performance optimization strategies at this point. A detailed code is publicly available at https://github.com/abreschi/shinySpecClust.

### Analysis

#### Feature generation

During clustering, each glycemic signature maintained an identifier noting the original participant and starting time. As such, glycemic signatures could easily be linked to participant, time, and glucose concentration. The portion of time spent in each class was estimated by calculating the fraction of a participant's windows assigned to the class. The frequency of each class was compared with age, BMI, HbA1c, fasting blood glucose, fasting insulin, fasting triglycerides, OGTT glucose concentration at 2 hours, insulin secretion rate, and SSPG.

In order to assess whether the clustering was meaningful, the glycemic signatures in each of the classes were compared using common metrics of variability in the CGM literature. These metrics included descriptive statistics measures such as mean/minimum/maximum glucose concentration, mean/maximum rate of change, interquartile range, and standard deviation. Other metrics included mean amplitude of glycemic excursion (MAGE), distance traveled, J index, and coefficient of variation [40, 41]. MAGE was calculated based on the Baghurst algorithm [42]. We note that using CGM, the classes, from low to severe, increase in both variability and mean glucose concentration, making them a more comprehensive metric of glycemic state compared to existing measurements.

#### Statistics

Forest plots and correlations were performed using a two-sided Pearson's product-moment correlation. Values reported are 95% confidence interval. A nonparametric Kruskal-Wallis rank sum test was used to compare the average values of common variability metrics for the glycemic signatures in each class. *P*-values are reported. Multiple hypothesis testing was performed using the Benjamini Hochberg method. A principal component analysis was performed to assess the separation by dominant glycemic signature class and current diagnostics. The features for the analysis included clinical tests and CGM metrics commonly used to assess blood sugar control: age, BMI, HbA1c, fasting blood sugar, OGTT at 2 hours, fasting insulin, high-sensitivity CRP, total cholesterol, triglycerides, high-density lipoprotein, low-density lipoprotein, mean glucose, standard deviation glucose, range of glucose, minimum glucose, maximum glucose, glucose 25% quantile, median glucose, glucose 75% quantile, mean rate of change, maximum range of change, number fluctuations above 140 mg/dL, number fluctuations above 200 mg/dL, percent readings below 80 mg/dL, percent readings above 130 mg/dL, standard error glucose mean, number glucose excursions above standard deviation, MAGE, J index, interquartile range, mean of daily differences at 6 AM, distance traveled, and coefficient of variation. The ranges of these values for individuals in the cohort are shown in S2 Table. SSPG was removed from the analysis because there were too many missing data. Diagnosis was determined based on the ADA cutoffs for HbA1c, OGTT at 2 hours, and fasting blood sugar [1]. Categorization of participants was based on the glycemic signature class in which they spent the largest portion of time.

#### Classification of standardized meal responses into glucotypes

The clustering into glucotypes was applied only to a subset of the CGM profiles for each participant (see Data preprocessing section); therefore, for several participants, not all standardized meals were covered by the selected portion of the CGM profile. Thus, we used the trained clustering to classify, or "predict," the entire profiles. To avoid analyzing some responses based on the initial classifications and others predicted after training, we recomputed the classes for the entire profiles and used those to assign the standardized meal responses to the three glycemic signatures L, M, and S. Consistently with our choice of parameters, we used sliding windows of 2.5 hours with a 75% overlap. We point out that "predicted" classes were only used for this analysis, while the rest of the study was based on the classified windows from the subset of the CGM profiles.

To classify the response to the standardized meals, we considered the windows starting in a 40-minute interval around the annotated consumption time of the meal, i.e., within 20 minutes before or 20 minutes after the meal. The responses were assigned to glucotypes based on the most severe glycemic signature of the overlapping windows.

## Supporting information

S1 Fig Parameter optimization for spectral clustering. Results of various clustering metrics for different combinations of parameters tested for spectral clustering (S2 Data). The rows of panels show the different clustering metrics, while the columns of panels are different types of overlaps used for generating the glycemic signatures. The y-axis for each panel shows the values of the clustering metrics in a given panel row. The x-axis shows the results for different distance metrics used in the clustering. The various colored lines show the results for different windows sizes tested in the analysis. CID, complexity invariant distance; DTW, dynamic time warping distance; variance, fraction of variance explained by the clustering; silhouette, average silhouette width for all glycemic signature; CH index, Calinski-Harabasz index.
(TIF)

S2 Fig Silhouette analysis for optimal spectral clustering. The silhouette width for each glycemic signature assigned to each class is plotted above (S3 Data and S4 Data). The panels split the signatures into each signature class shown in Fig 2. The x-axis shows the actual silhouette width, with positive and negative values indicating a good or bad fit for that glycemic signature in that class. Positive silhouette values indicate that the glycemic signatures are good fits for their given class, while negative silhouettes indicate that a given glycemic signature is not a good fit for its class. A value of 1 indicates a perfect fit.
(TIF)

S3 Fig Pairwise Spearman's correlation coefficients between clinical variables, CGM-derived variability measures, and frequency of glucotype windows. (S5 Data). CGM, continuous glucose monitoring.
(TIF)

S4 Fig Glycemic response to standardized meals. The interstitial glucose concentrations from 30 minutes prior to the start of each meal until 2.5 hours after the start of each meal are shown (S6 Data). Each line represents a unique response from a participant. The three panels separate the responses by type of standardized meal eaten. Note that each individual has 0 to 2 responses shown, depending on how many times they ate each meal.
(TIF)

S1 Table Cohort characteristics. (TIF)

S2 Table Cohort characteristics split by ADA diagnosis. Participant clinical characteristics are shown in the table above split by diagnosis. This diagnosis was based on ADA Guidelines of HbA1c, fasting blood sugar, and 2hr OGTT. The table displays the mean and standard deviation for the entire cohort and subsets of the cohort. Units for the clinical and laboratory results in the table are as follows: age in years; SSPG, FBG, and OGTT all in mg/dL glucose concentration; fasting insulin in mIU/L; HbA1c in percent blood concentration; hsCRP in mg/L; TriHDL is unitless. All sensor and glucose variability metrics are listed in mg/dL interstitial glucose concentration with the excepted of the following: mean and maximum slope are in mg/dL/min; coefficient of variation and number of fluctuations are unitless. 2hr OGTT, blood glucose concentration 2 hours after the start of oral glucose tolerance test; ADA, American Diabetes Association; BMI, body mass index; FBG, fasting blood glucose; hsCRP, high-sensitivity C-reactive protein; LDL/HDL, high- and low-density lipoprotein; SSPG, steady-state plasma glucose.
(TIF)

S3 Table Comparison of glucotypes by common CGM metrics. Mean values of common metrics of glycemic variability for each of the classes shown in Fig 2. The metrics are calculated for each window. A Kruskal-Wallis multiple ANOVA test was performed to determine whether these values differed significantly between glycemic signature classes. The resulting *p*-value was significant for all metrics tested (<1e – 100). CGM, continuous glucose monitoring.
(TIF)

S4 Table Strength of correlation of current variability metrics and glucotype with clinical metrics. Above is a table examining the correlation common CGM variability metrics and glucose homeostasis. The first three rows are the three classes of glycemic signatures. The last several rows are common measures of glucose variability used in CGM analysis. The analysis correlated either the value of these measures or the fraction of time spent in each of these classes with clinical tests using a Pearson's correlation test. Shown are the resulting correlation coefficients with notes to indicate the significance of the *p*-values. (significance codes: 0 = “***”, 0.001 = “**”, 0.01 = “*”, 0.05 = “.”). CGM, continuous glucose monitoring; coef_variation, coefficient of variation; IQR, interquartile range; j_index, J index; mage, mean amplitude of glycemic response; OGTT—2hr, blood glucose concentration 2 hours after the start of oral glucose tolerance test; sd_glucose and mean_glucose, standard deviation and mean glucose concentration; SSPG, steady-state plasma glucose; triglycerides/HDL, triglyceride concentration divided by high-density lipoprotein concentration, an approximation of insulin resistance.
(TIF)

S5 Table Nutrition facts for standardized meals. The table displays the nutritional content of each of the standardized meals used for the study. Values are for the entire meal, and units are listed in grams, except calories, which are listed as kilocalories.
(TIF)

S1 Data Continuous glucose monitor recordings. Glucose concentration recorded every 5 minutes. Columns are DisplayTime (time stamp of recording as shown by the monitor, used for analysis), GlucoseValue (glucose concentration in mg/dl), subjectId (participant id), and InternalTime (internal time stamp of recording).
(GZ) Click here for additional data file.

S2 Data Parameters tested and clustering metrics. Clustering metrics for different combinations of parameters tested. The metrics are reported for the optimal number of clusters as identified by the eigengap statistics. Columns are distance (distance metric), shift (minutes sliding windows are shifted, corresponding to the percentage of overlap), window_size_hrs (window size in hours), clust_metric (clustering metric type), value (value for the corresponding clustering metric), and window_overlap_perc (percentage of window overlap).
(TSV)

S3 Data Glycemic signature class. Assignment of glycemic signature class to each window of 2.5 hours and 75% overlap. Columns are window id, class. The window id is built as subject id + window size (hours) + shift (minutes) + window start time.
(TSV)

S4 Data Silhouette values. Silhouette values for all windows after spectral clustering. Columns are window id, silhouette.
(TSV)

S5 Data Glucotype and clinical variables. Master table with participants' information and clinical variables in the format of SQLite database. It also contains the frequency of windows into each class (low, moderate, severe), as well as the resulting glucotype assigned to each participant.
(DB) Click here for additional data file.

S6 Data Glucose concentration after standardized meals. Glucose concentration values from 30 minutes before the standardized meal and 2.5 hours after. Columns are Meal (meal consumed), userID (participant id), time (time stamp for glucose concentration), GlucoseValue (glucose concentration in mg/dl).
(TSV)

S7 Data Classification of glycemic response to standardized meals. Glycemic signature class assigned to the response to each standardized meal. Columns are userID (participant id), meal (id for standardized meal), glucotype, nb_meals_glucotype (glucotype assigned to a meal for each participant), mealType (meal type: protein bar, corn flakes, bread with peanut butter), nb_meals_tot (number of standardized meal each participant had in total).
(TSV)

S8 Data Insulin secretion rate. Insulin secretion rate estimated from C-peptide concentration during OGTT. Columns are userID (participant id), date (date the test was taken), timepoints_min (minutes along the test), value (value for parameter), parameter. Parameters include Calculated_C (estimated C-peptide concentration, pmol/l), Measured_C (measured C-peptide concentration, pmol/l), Insulin_uuml (insulin, mlU/ml), insulin (fasting insulin, mlU/ml), Insulin_Rate (insulin secretion rate, pmol/kg/min), FBG (mg/dl), OGTT (blood glucose concentration during OGTT, mg/dl), SSPG (plasma glucose concentration after SSPG test, mg/dl). FBG, fasting blood glucose; OGTT, oral glucose tolerance test; SSPG, steady-state plasma glucose.
(TSV)

## Abbreviations

ADA: American Diabetes Association
CGM: continuous glucose monitoring
CH: Calinski-Harabasz
CID: complexity-invariant distance
CID-DTW: complexity-invariant dynamic time warping
CTRU: Clinical Translational Research Unit
DTW: dynamic time warping
ISEC: Insulin SECretion
MAGE: mean amplitude of glycemic excursion
OGTT: oral glucose tolerance test
SSPG: steady-state plasma glucose
SSPI: steady-state plasma insulin
totBSS: total between-cluster sum of square
totSS: total sum of square
