# Supplementary figures and images for: Glucotypes reveal new patterns of glucose dysregulation
Source: PLoS Biol. 2018 Jul 24;16(7):e2005143. doi: 10.1371/journal.pbio.2005143 (PMC6057684; doi:10.1371/journal.pbio.2005143)

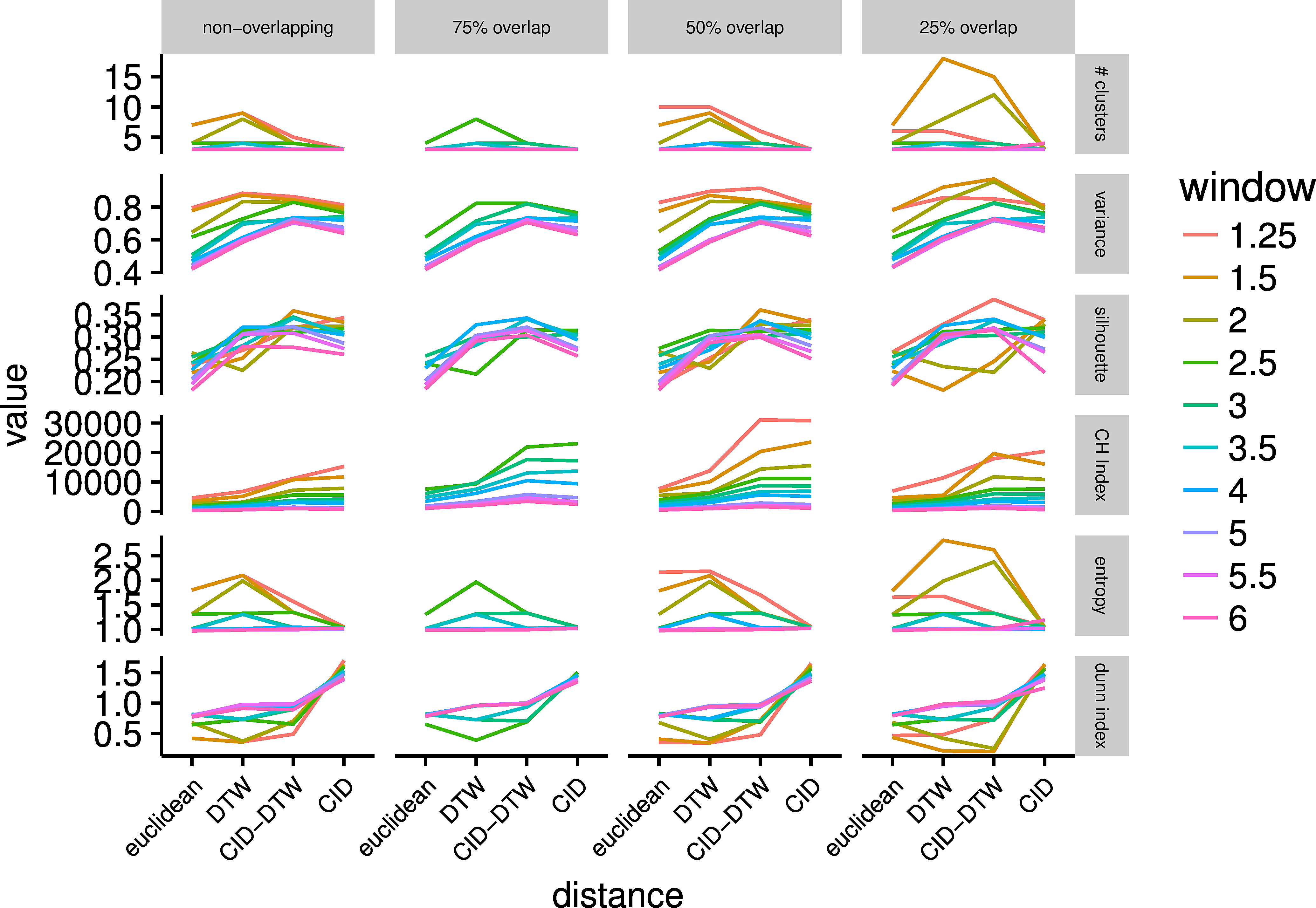

Supplement: S1 Fig — Results of various clustering metrics for different combinations of parameters tested for spectral clustering (S2 Data). The rows of panels show the different clustering metrics, while the columns of panels are different types of overlaps used for generating the glycemic signatures. The y-axis for each panel shows the values of the clustering metrics in a given panel row. The x-axis shows the results for different distance metrics used in the clustering. The various colored lines show the results for different windows sizes tested in the analysis. CID, complexity invariant distance; DTW, dynamic time warping distance; variance, fraction of variance explained by the clustering; silhouette, average silhouette width for all glycemic signature; CH index, Calinski-Harabasz index. (TIF) [file pbio.2005143.s001.tif]

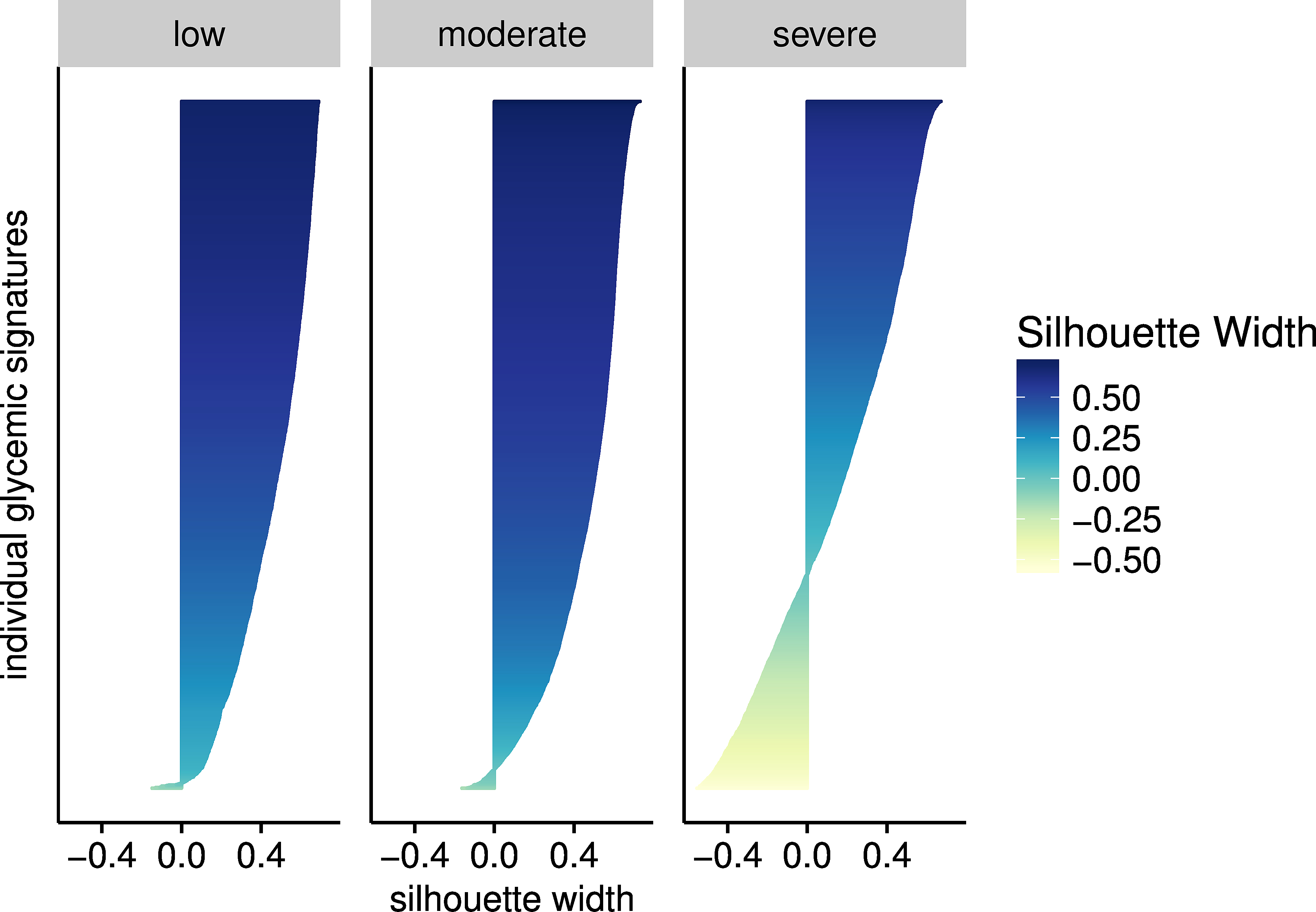

Supplement: S2 Fig — The silhouette width for each glycemic signature assigned to each class is plotted above (S3 Data and S4 Data). The panels split the signatures into each signature class shown in Fig 2. The x-axis shows the actual silhouette width, with positive and negative values indicating a good or bad fit for that glycemic signature in that class. Positive silhouette values indicate that the glycemic signatures are good fits for their given class, while negative silhouettes indicate that a given glycemic signature is not a good fit for its class. A value of 1 indicates a perfect fit. (TIF) [file pbio.2005143.s002.tif]

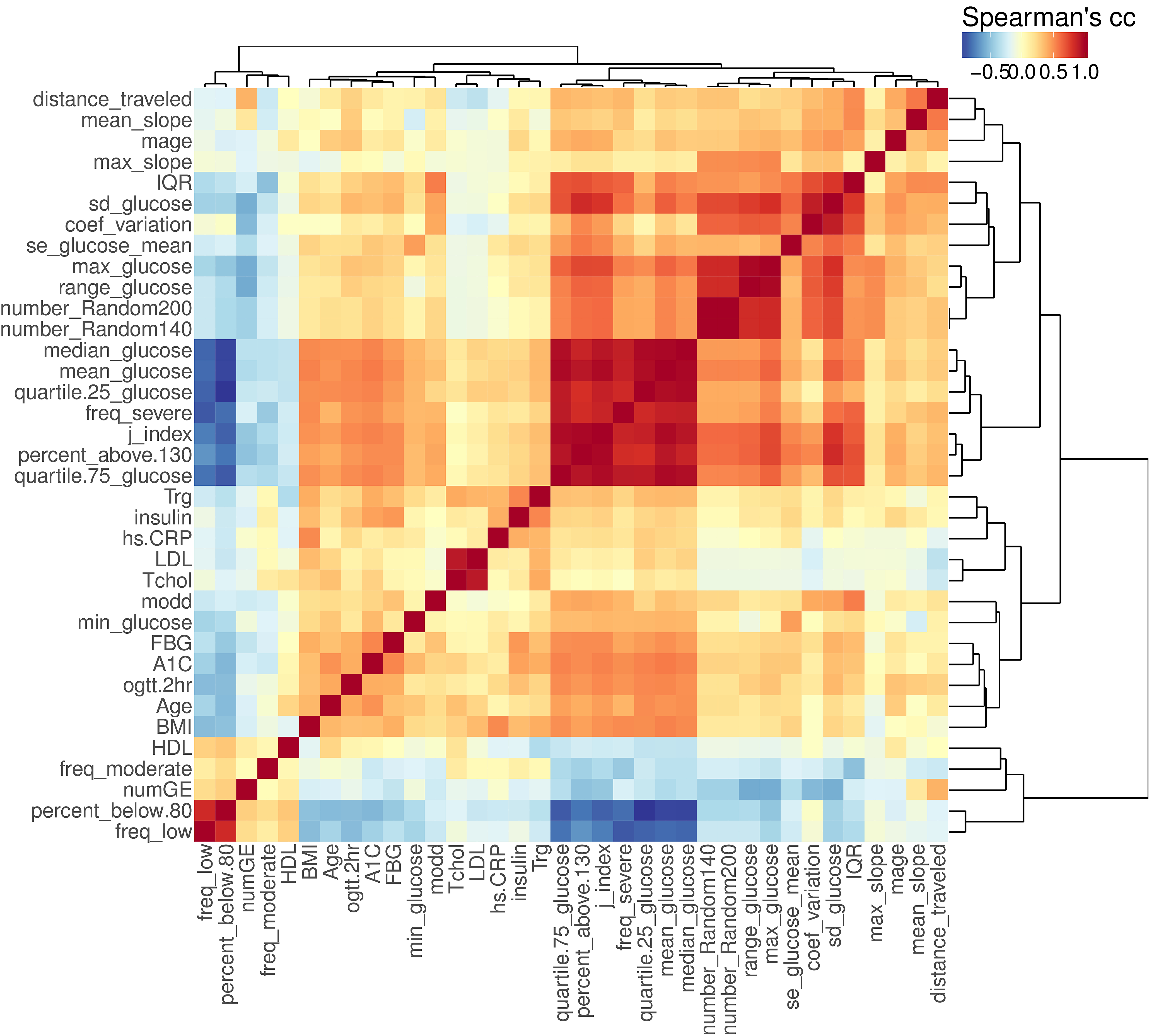

Supplement: S3 Fig — (S5 Data). CGM, continuous glucose monitoring. (TIF) [file pbio.2005143.s003.tif]

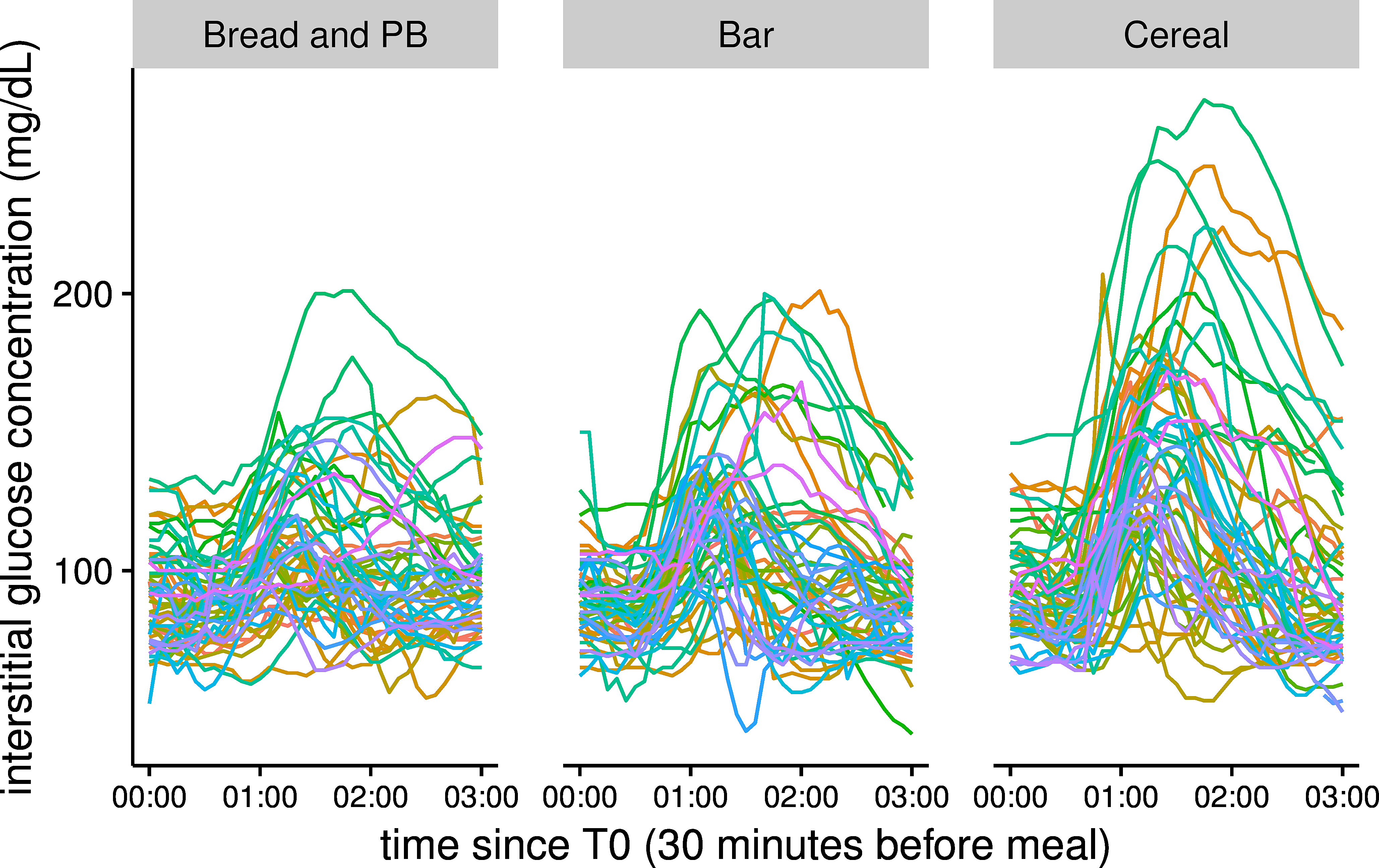

Supplement: S4 Fig — The interstitial glucose concentrations from 30 minutes prior to the start of each meal until 2.5 hours after the start of each meal are shown (S6 Data). Each line represents a unique response from a participant. The three panels separate the responses by type of standardized meal eaten. Note that each individual has 0 to 2 responses shown, depending on how many times they ate each meal. (TIF) [file pbio.2005143.s004.tif]

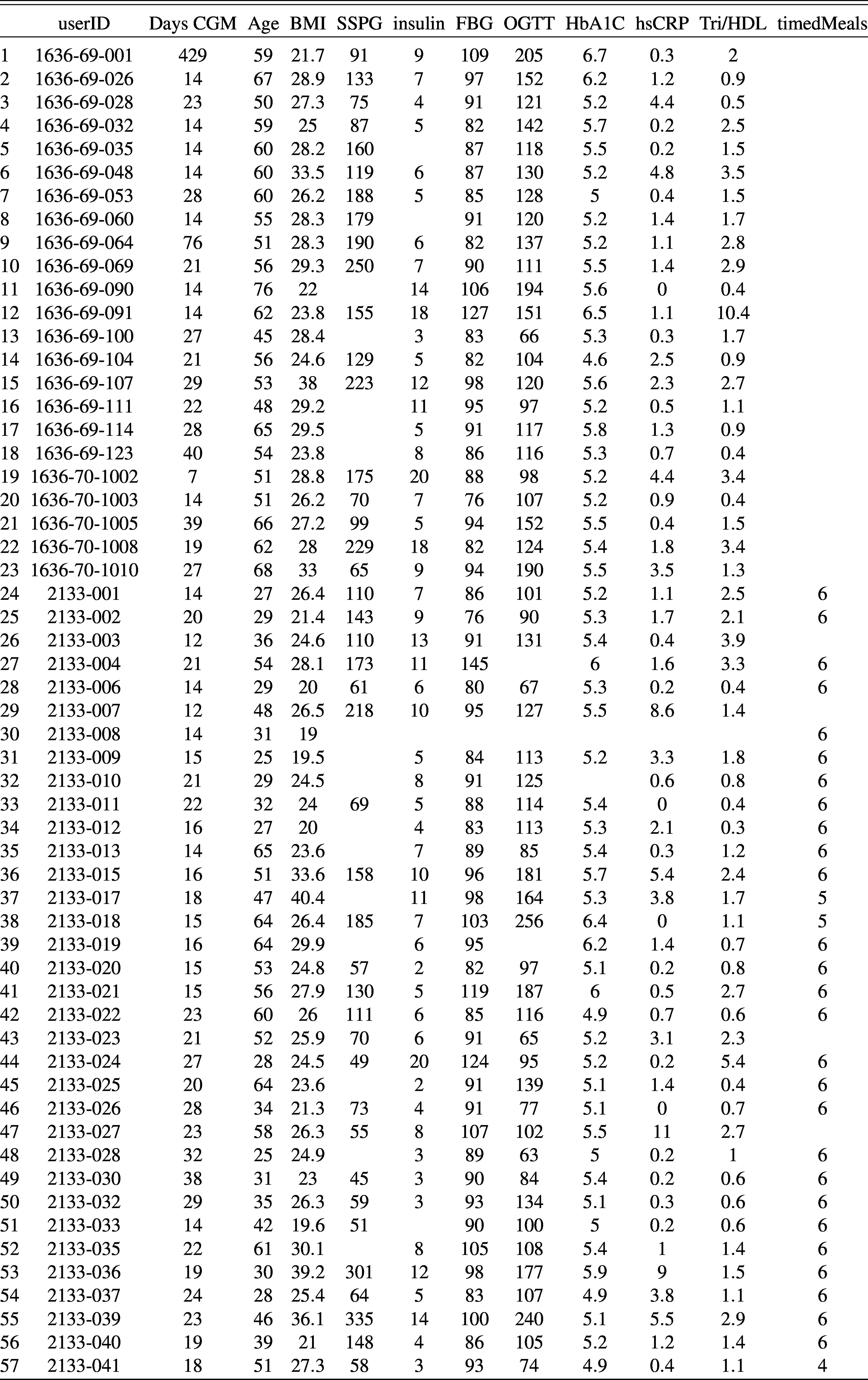

Supplement: S1 Table — (TIF) [file pbio.2005143.s005.tif]

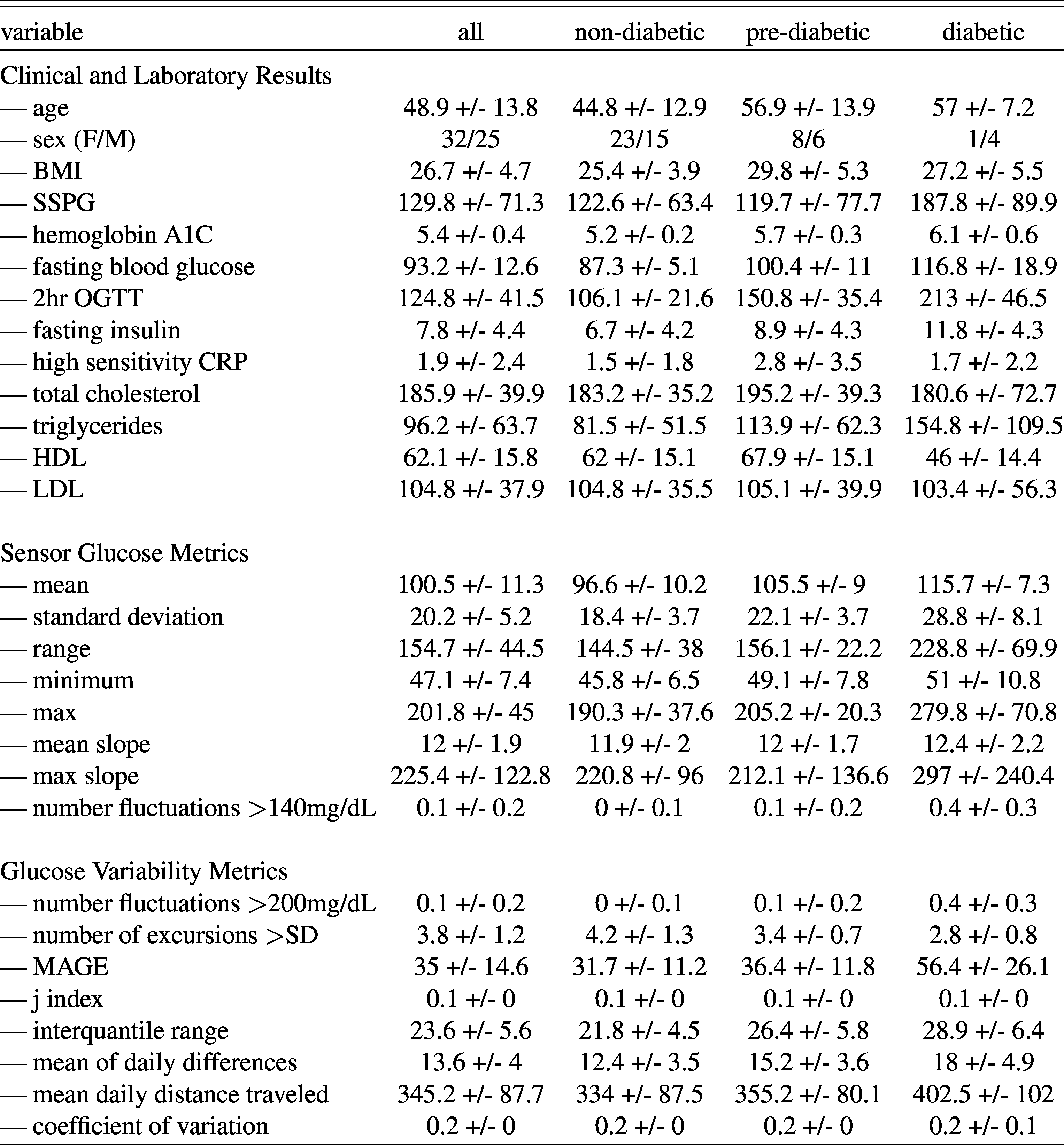

Supplement: S2 Table — Participant clinical characteristics are shown in the table above split by diagnosis. This diagnosis was based on ADA Guidelines of HbA1c, fasting blood sugar, and 2hr OGTT. The table displays the mean and standard deviation for the entire cohort and subsets of the cohort. Units for the clinical and laboratory results in the table are as follows: age in years; SSPG, FBG, and OGTT all in mg/dL glucose concentration; fasting insulin in mIU/L; HbA1c in percent blood concentration; hsCRP in mg/L; TriHDL is unitless. All sensor and glucose variability metrics are listed in mg/dL interstitial glucose concentration with the excepted of the following: mean and maximum slope are in mg/dL/min; coefficient of variation and number of fluctuations are unitless. 2hr OGTT, blood glucose concentration 2 hours after the start of oral glucose tolerance test; ADA, American Diabetes Association; BMI, body mass index; FBG, fasting blood glucose; hsCRP, high-sensitivity C-reactive protein; LDL/HDL, high- and low-density lipoprotein; SSPG, steady-state plasma glucose. (TIF) [file pbio.2005143.s006.tif]

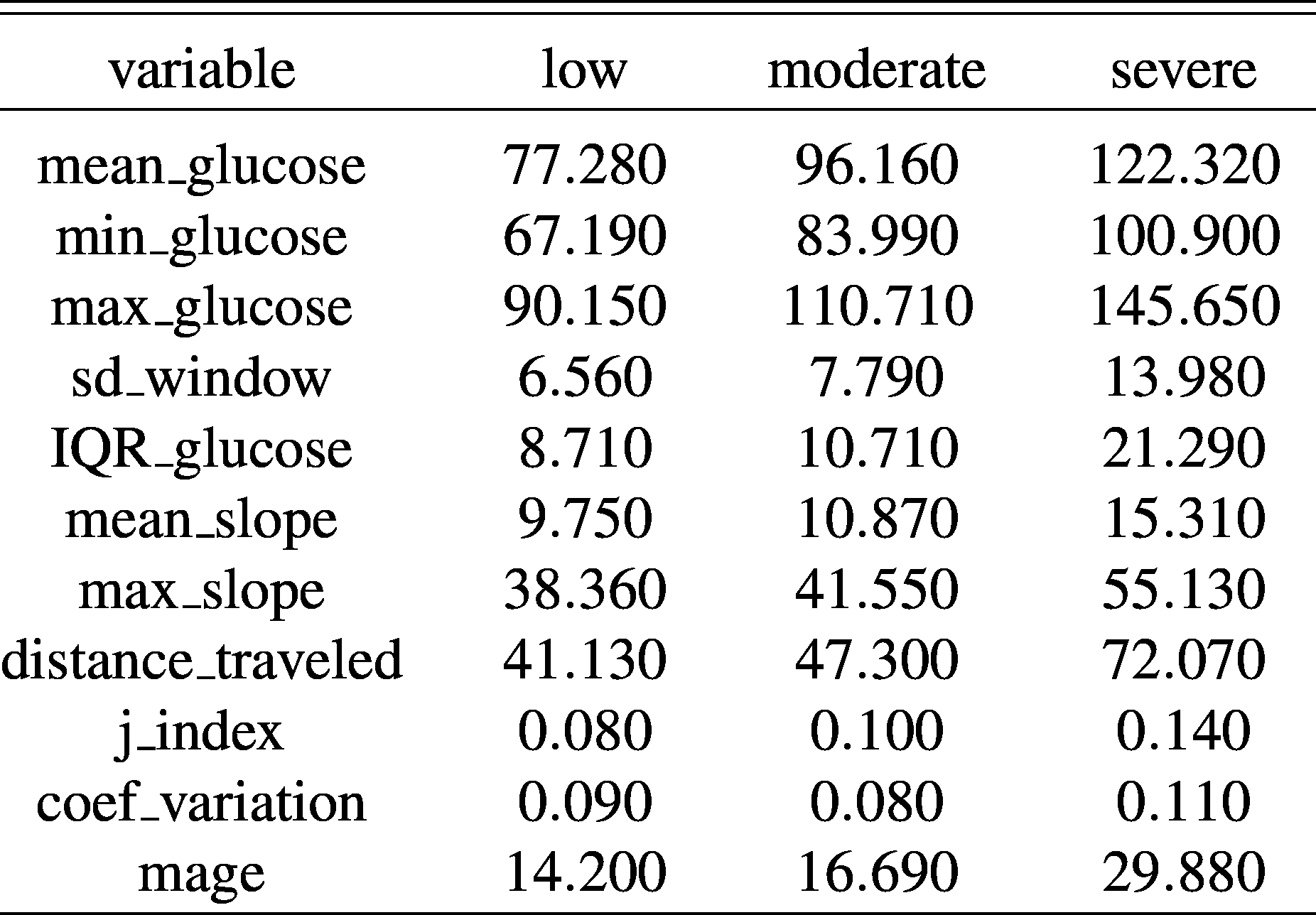

Supplement: S3 Table — Mean values of common metrics of glycemic variability for each of the classes shown in Fig 2. The metrics are calculated for each window. A Kruskal-Wallis multiple ANOVA test was performed to determine whether these values differed significantly between glycemic signature classes. The resulting p-value was significant for all metrics tested (<1e – 100). CGM, continuous glucose monitoring. (TIF) [file pbio.2005143.s007.tif]

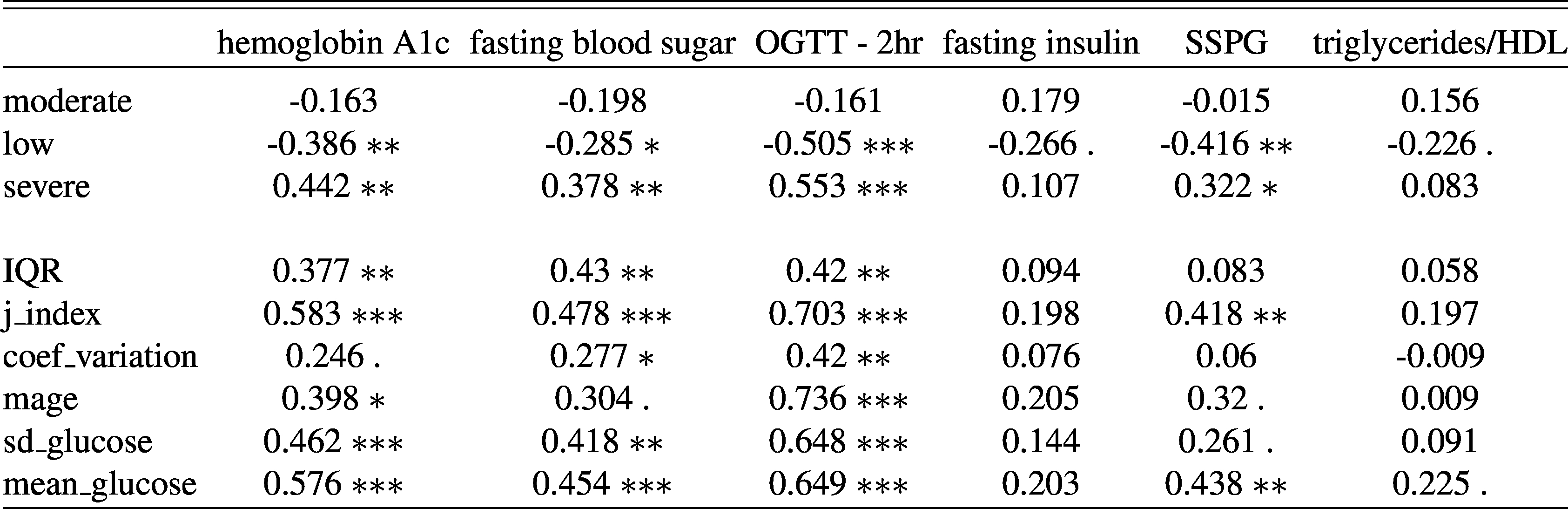

Supplement: S4 Table — Above is a table examining the correlation common CGM variability metrics and glucose homeostasis. The first three rows are the three classes of glycemic signatures. The last several rows are common measures of glucose variability used in CGM analysis. The analysis correlated either the value of these measures or the fraction of time spent in each of these classes with clinical tests using a Pearson's correlation test. Shown are the resulting correlation coefficients with notes to indicate the significance of the p-values. (significance codes: 0 = “***”, 0.001 = “**”, 0.01 = “*”, 0.05 = “.”). CGM, continuous glucose monitoring; coef_variation, coefficient of variation; IQR, interquartile range; j_index, J index; mage, mean amplitude of glycemic response; OGTT—2hr, blood glucose concentration 2 hours after the start of oral glucose tolerance test; sd_glucose and mean_glucose, standard deviation and mean glucose concentration; SSPG, steady-state plasma glucose; triglycerides/HDL, triglyceride concentration divided by high-density lipoprotein concentration, an approximation of insulin resistance. (TIF) [file pbio.2005143.s008.tif]

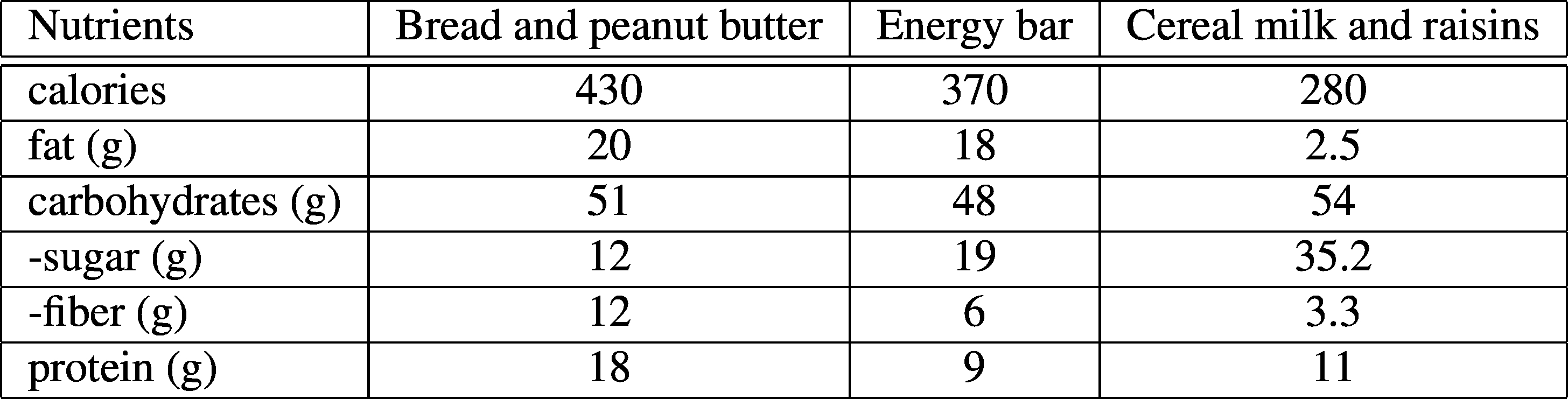

Supplement: S5 Table — The table displays the nutritional content of each of the standardized meals used for the study. Values are for the entire meal, and units are listed in grams, except calories, which are listed as kilocalories. (TIF) [file pbio.2005143.s009.tif]
